# Study on nonlinear dynamic characteristics of a two-speed transmission system at low speed

**DOI:** 10.1371/journal.pone.0298395

**Published:** 2024-02-14

**Authors:** Liu Zhihui, Jiahao Zhang, Zhijian Zhang, Yingzhi Gu, Xue Wen, Kejun Zhu

**Affiliations:** 1 College of Mechanical and Vehicle Engineering, Hunan University, Changsha, China; 2 College of Mechanical and Energy Engineering, Shaoyang University, Shaoyang, China; 3 Hunan Provincial Key Laboratory of Intelligent Manufacturing of High Efficiency Power System, Shaoyang University, Shaoyang, China; Wroclaw University of Science and Technology: Politechnika Wroclawska, POLAND

## Abstract

A pure shear mechanical model of low gear of six-degree-of-freedom two-speed transmission system is established by using lumped parameter method. The Runge-Kutta method is used to numerically solve the aforementioned nonlinear system. The variation of transmission error between gears is analyzed by using global bifurcation, time domain diagram, phase diagram and Poincare cross section. Moreover, the transfer error bifurcation characteristics of the solar wheel and the first planetary wheel under different gear moduli are investigated. The results show that: by taking the excitation frequency as the control parameter, the system includes period-1 motion, period-2 motion, quasi-periodic motion, multiperiodic motion, and chaotic motion. With the increase of gear modulus, the system mainly presents chaotic motion in the medium frequency range (0.5<ω_*h*_≤2). It shows stable period-1 motion in the high frequency range (2<ω_*h*_≤3), and the higher the modulus, the wider the high frequency range of period-1 motion. The research results can provide reference for the design and optimization of this kind of two-speed transmission system in the future.

## Introduction

The two-speed transmission system can achieve two-speed output and is used in helicopters, tanks, loaders, automobiles, and other fields. The two-speed transmission system is adopted in the main reducer of the helicopter, thereby achieving cruise and hovering of the helicopter (starting and stopping). Consequently, fuel consumption can be lowered. A typical structure of a two-speed transmission system consists of a friction clutch, an overrunning clutch, and a planetary gear transmission system [1–3]. The overrunning clutch is in the overrun state and the system output is a high-speed gear output when the control friction clutch is combined; the opposite is true when the control friction clutch is released and the overrunning clutch is in the combined state and the system output is a low-speed output. The dynamic characteristics analysis of a two-speed transmission system is separated into three categories: shift process analysis, low-speed gear analysis, and high-speed gear analysis. This study primarily examines the low-speed transmission characteristics of a two-speed transmission system. The study of the two-speed transmission system’s nonlinear dynamic characteristics can serve as a foundation for the best possible design and manufacturing of the transmission system. The two-speed transmission system’s operational performance has a direct impact on the functionality and lifespan of helicopters and other equipment.

During low-speed transmission, the overrunning clutch and the planetary gear transmission system form a coupling system. Nonlinear characteristics of the planetary gear transmission system are mainly investigated in three aspects: modeling method, solution method, and stability evaluation. The models mainly include pure torsional and bending-torsion coupling models. The time-varying meshing stiffness, lateral clearance, and comprehensive meshing error of the gear teeth are usually considered during modeling. Wei et al. [4] proposed a method to establish the dynamic model of the planetary gear system by using shafting elements such as shaft segments, flexible ring gear, and flexible planetary carriers. This method can guide the design of the planetary gear system with high reliability and low vibration. Huang et al. [5] proposed a nonlinear pure rotational dynamics model for a multistage closed planetary gear set consisting of two simple planetary stars. Gui et al. [6] developed a centralized parameter model for a typical planetary gear system with different error types.

Analytical and numerical methods are typically employed for solving the nonlinear dynamics of a planetary gear transmission system. The analytical methods typically include multi-scale, harmonic balance, modal superposition, and Fourier series. Numerical methods include the iterative, Euler, Runge-Kutta, and Gill methods. Lin et al. [7] applied the multi-scale method to investigate the parameter conditions of an unstable planetary gear system. Chaari et al. [8] used an iterative method to calculate the nonlinear dynamic response of the planetary gear system. Zhang et al. [9] solved the vibration response of the 2K-H planetary gear reducer with planetary suspension by employing the Fourier series method. Moreover, the authors analyzed the influence of gear error, planetary suspension, and the initial meshing phase on the dynamic characteristics of the system. Sun et al. [10, 11] analyzed the dynamic characteristics of a closed planetary gear system under the dynamic coupling of the star gear train and planetary gear train by utilizing the Gill integral method with variable step length. Wu et al. [12] studied the nonlinear dynamic characteristics of the compound planetary wheel drive system based on the harmonic balance method. Xu et al. [13] established a new gear tooth modification model according to tooth top modification characteristics and tooth profile modification of the planetary gear train.

Dynamic stability and motion state research on a planetary gear transmission system is mainly based on the nonlinear dynamic model of the system. The numerical algorithm is used to study the system’s stability and possible motion state under different parameters. Xiang et al. [14] analyzed the planetary gear system’s motion and various nonlinear dynamics via the global bifurcation diagram, FFT spectrum, Poincare diagram, phase diagram, and maximum Lyapunov exponent. Zhou et al. [15] explored the reliability of the shearer planetary gear system and the sensitivity analysis based on reliability. The results show that the structural parameters of the solar wheel significantly impact the system’s reliability compared to other mechanisms. Li et al. [16] established the reliability prediction model of a helicopter planetary gear train when subjected to partial loading. Xiang et al. [17] identified the influence of system motion on backlash variation by using a global bifurcation diagram, maximum Lyapunov exponent (LLE), FFT spectrum, Poincare diagram, phase diagram, and time series. Zhou et al. [18] analyzed the effects of meshing frequency, meshing damping, and tooth clearance on the bifurcation and chaos characteristics of the system via phase orbits, Poincare plots, and time history curves.

Kumar [19] proposed a deep learning model and tested the proposed deep learning model on both gear and rotor datasets. The result is more efficient than the current method. Vashishtha [20] proposed an optimal selection of hyperparameters (HPs) based on a deep learning model to study worm gearboxes and found that this method is more efficient than conventional methods. Zhou [21] proposed a health index prediction method based on discrete probabilistic entropy and a bearing health prediction method based on long and short term memory, and the comparison shows that the method is superior to other time series prediction models. Vashishtha [22] proposed an intelligent defect identification scheme for tapered roller bearings based on the ELM model. The original vibration signal of bearing test bench is decomposed into different modes to remove noise. Permutation entropy (PE) is used as a measure to select the prominent pattern. Good experimental results are obtained. Vashishtha [23] proposed a deep learning-based bearing fault identification method. The results show that the language fuzzy classifier achieves the maximum performance with the least computation time. Vashishtha [24] proposes a new method for identifying defects in centrifugal pump impellers, and the end result is improved system reliability ACMD performance.

From the above analysis, it can be seen that there have been many studies on the nonlinear characteristics of a pure planetary gear transmission system but less research on two-speed transmission, especially the dynamic characteristics of the coupling system composed of an overrunning clutch and gear transmission system. The overrunning clutch and the gear drive system inevitably influence each other. Therefore, in order to explore the vibration mechanism of low-speed gear in a two-speed transmission system, this paper intends to use the centralized parameter method to establish a system of pure torsional nonlinear dynamic differential equations considering the number of planetary wheels, tooth side clearance, and gear transmission error for the low-speed gear in the two-speed transmission system. The variable-step fourth-order Runge-Kutta method is used to solve the dimensionless differential equation system, and the influence of gear modulus and other parameters on the nonlinear dynamic characteristics of the two-speed transmission system is analyzed by taking the excitation frequency as the control parameter. The research results can provide a reference for the dynamic design and optimization of such two-speed transmission systems in the future and provide theoretical guidance for the mobility and economy of two-speed transmission systems.

## Structure composition and working principle of a two-speed transmission system

**Fig 1:**
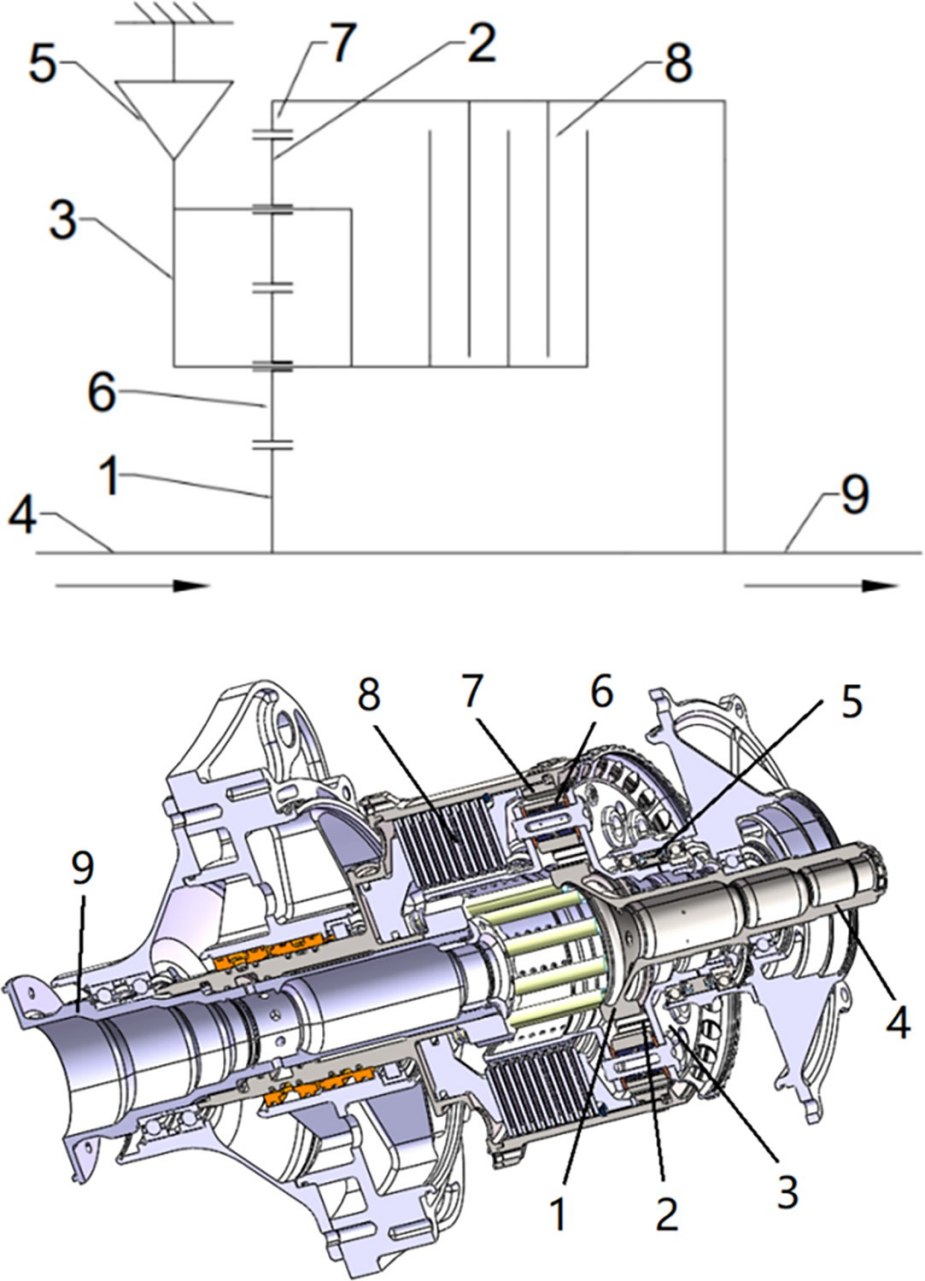
A two-speed drivetrain: (a) Schematic with (1) the sun wheel, (2) the first stage planetary wheel, (3) the planetary shelf, (4) the input axis, (5) the overrunning clutch, (6) the second stage planetary wheel, (7) the inner gear ring, (8) the friction clutch, and (9) the output shaft; (b) 3D model.

A two-speed transmission system shown in Fig 1 comprises the input shaft, double-row planetary gear of the planetary gear train, friction clutch, overrunning clutch, and output shaft structure. The overrunning clutch is set between the planet carrier and the rack; the friction clutch is set between the planet carrier with the output shaft, power input by the input shaft, and output by the output shaft. When the friction clutch is released, the overrunning clutch is in the engaged state, and the planetary rack is in the fixed state. The power is transmitted through the sun wheel, a double row of planetary wheels, and finally, the inner gear ring output, which corresponds to the low gear state. When the friction clutch is engaged, the overrunning clutch is overrunning, and the planetary rack is connected with the inner gear ring. The power is also successively passed through the sun wheel, a double row of planetary wheels, and the inner gear ring output. The output shaft is at a high speed, corresponding to the high-speed stop state.

A certain type of two-speed transmission system is applied to the helicopter. During 70%–80% of the working time, the transmission system is in the low-speed gear state, while the double-row planetary wheels are stationary in the high-speed gear state. Therefore, the focus is placed on the dynamic characteristics of the low-speed gear state in this paper.

### Low-speed transmission dynamics model of a two-speed transmission system

**Fig 2:**
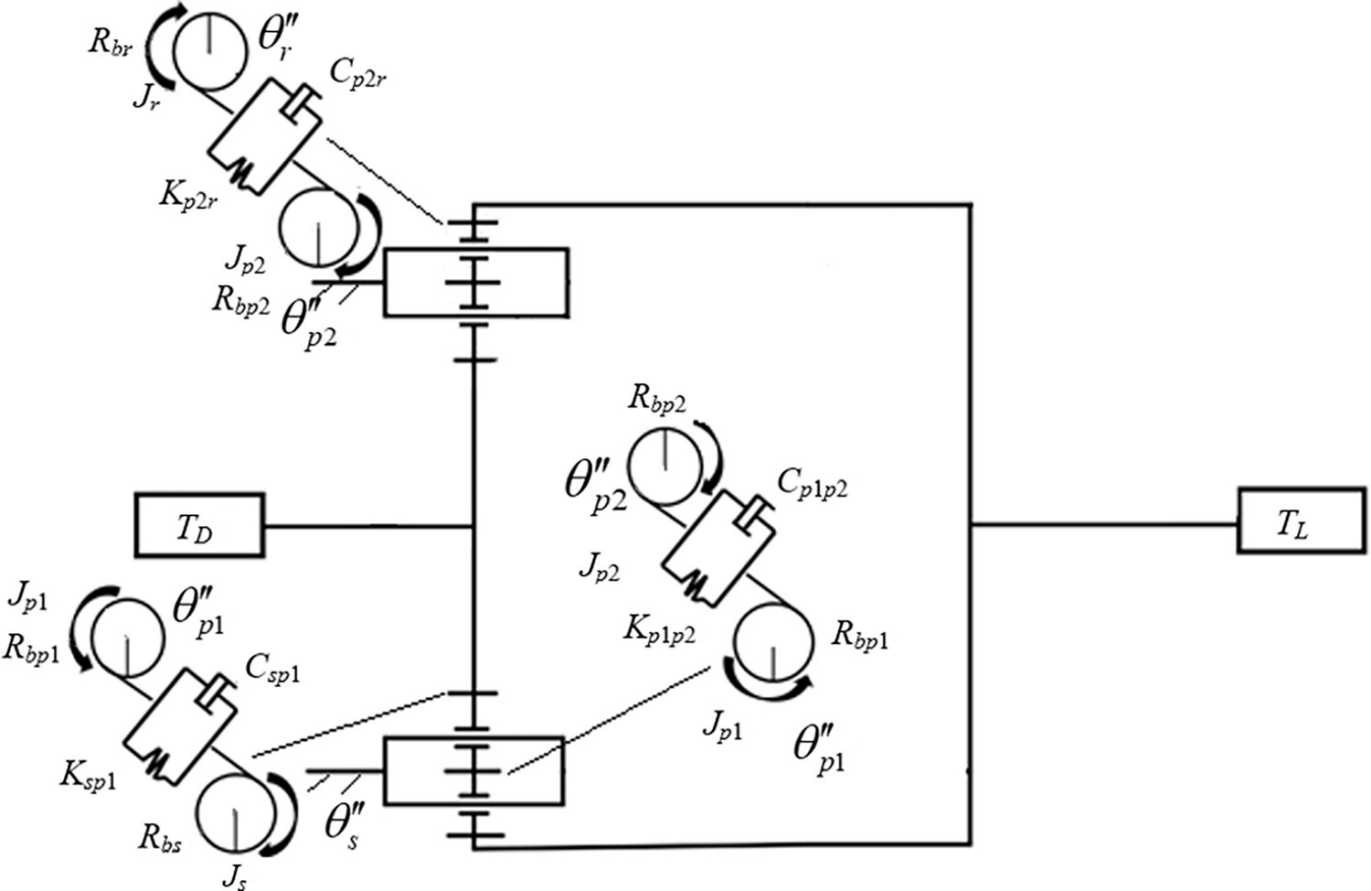
Dynamics model of a two-speed transmission system in low gear.

The centralized mass method is adopted to establish the dynamic model of a two-speed transmission system running in low gear, as shown in Fig 2. The model takes the planetary frame as the reference coordinate system and the locked state. The fixed shaft transmission is the planetary gear system with double rows of planetary wheels. The three degrees of freedom of the solar wheel were fixed on the planetary shelf, and the origin was placed in the center of the planetary shelf. The coordinate system origin of the double row of planetary wheels is taken as the center of each planetary wheel, which is also fixed on the planetary shelf.

In Fig 2, *R*_*bs*_ represents the radius of the base circle of the solar wheel; *R*_*bp*1_ is the base circle radius of the first stage planetary gear; *R*_bp2_ is the base circle radius of the second stage planetary gear; *R*_*br*_ is the radius of the base circle of the inner gear ring; *T*_*D*_ is the driving torque; *T*_*L*_ is the load torque. *K*_sp1_ is the time-varying meshing stiffness of the solar wheel and the first planetary wheel; *C*_sp1_ is the meshing damping of the solar wheel and the first stage planetary wheel; *K*_p1p2_ is the time-varying meshing stiffness of the second stage planetary wheel and the first stage planetary wheel; *C*_p1p2_ is the meshing damping of the second stage planetary wheel and the first stage planetary wheel; *K*_p2r_ is the time-varying meshing stiffness of the inner gear ring and the second planetary wheel; *C*_p2r_ is the meshing damping between the inner ring gear and the second stage planetary wheel. Parameters $\theta_{s}^{\prime \prime },\;\theta_{p1}^{\prime \prime },\;\theta_{p2}^{\prime \prime }$, and $\theta_{r}^{\prime \prime }$ are angular accelerations of the sun wheel, the first planetary wheel, the second planetary wheel, and the inner gear ring, respectively. Parameters *J*_*s*_, *J*_*p*1_, *J*_*p*2_, and *J*_*r*_ are the inertia of the sun wheel, the first planetary wheel, the second planetary wheel, and the inner gear ring, respectively.

## Low gear dynamics equation of two-speed transmission system

As shown in Fig 2, *K*_*sp*1_(t) is the time-varying meshing stiffness of the solar wheel and the first planetary gear pair. Its value can be regarded as a rectangular wave, as shown in Eqs 1−4:

$$k_{sp1}(t)=k_{sp1}(t+2\pi /\omega )=k_{sp1}+\sum_{r=1}^{R}k_{sp1r}\cos(r\omega_{e}t‐\varphi_{r}),$$ (1)

$$k_{sp1}/k_{tp}=\varepsilon_{sp1},$$ (2)

$$k_{sp1r}/k_{tp}=\sqrt{2-2\cos(2\pi r(\varepsilon_{sp1}-1))}/\left(\pi r\right),$$ (3)

$$\varphi_{sp1r}=a\tan(\left(1-\cos(2\pi r(\varepsilon_{sp1}-1))\right)/\left(\sin(2\pi r(\varepsilon_{sp1}-1))\right)),$$ (4)

Where *k*_*sp*1_ is the average value of time-varying meshing stiffness; *k*_*sp*1*r*_ is the RTH harmonic amplitude; *φ*_*sp*1*r*_ is the phase angle of the RTH harmonic; *ε*_*sp*1_ is the coincidence degree of the solar wheel and the first stage planetary gear. The R-value is taken as five since the first five harmonics have relatively accurate accuracy.

It is assumed that all gears are unmodified involute spur gears, the bending deformation of input and output shafts is neglected, and the centralized parameter method and Newton’s law are employed. Then, the pure torsional nonlinear mathematical equation of the two-speed transmission system can be expressed as follows:

$$\left\{ \begin{array}{l} \sum_{i=1}^{N}(C_{sp1i}(R_{bs}{\dot{\theta }}_{s}+R_{bp1i}{\dot{\theta }}_{p1i}-{\dot{e}}_{sp1i}(t))R_{bs}+K_{sp1i}(R_{bs}\theta_{s}+R_{bp1i}\theta_{p1i}\\ -e_{sp1i}(t))R_{bs})+J_{S}{\ddot{\theta }}_{S}=T_{D}(t)\\ C_{sp1i}(R_{bs}{\dot{\theta }}_{s}+R_{bp1i}{\dot{\theta }}_{p1i}-{\dot{e}}_{sp1i}(t))R_{p1i}+C_{p1ip2i}(R_{bp1i}{\dot{\theta }}_{p1i}+\\ R_{bp2i}{\dot{\theta }}_{p2i}-{\dot{e}}_{p1ip2i}(t))R_{bp1i}+K_{p1ip2i}(R_{bp1i}\theta_{p1i}+R_{bp2i}\theta_{p2i}-e_{p1ip2i}(t))\\ R_{bp1i}+K_{sp1i}((R_{bs}\theta_{s}+R_{bp1i}\theta_{p1i}-e_{sp1i}(t))R_{bp1i}+J_{p1i}{\ddot{\theta }}_{p1i}=0\\ C_{p1ip2i}(R_{bp1i}{\dot{\theta }}_{p1i}+R_{bp2i}{\dot{\theta }}_{p2i}-{\dot{e}}_{p1ip2i}(t))R_{p2i}-C_{p2ir}(R_{br}{\dot{\theta }}_{r1}R_{bp2i}\\ -R_{bp2i}{\dot{\theta }}_{p2i}-{\dot{e}}_{p2ir}(t))R_{bp2i}+K_{p1ip2i}((R_{bp1i}\theta_{p1i}+R_{bp2i}\theta_{p2i}-e_{p1ip2i}(t))\\ R_{bp2i}-K_{p2ir}(R_{br}\theta_{r1}-R_{bp2i}\theta_{p2i}-e_{p2ir1}(t))R_{bp2i}+J_{p2i}{\ddot{\theta }}_{p2i}=T_{L} \end{array}\right.$$ (5)

Where *θ*_*s*_, *θ*_*p*1*i*_, *θ*_*p*2*i*_, *θ*_*r*1_ are torsional vibration displacements of the sun wheel, the ith planetary wheel of the first stage, the ith planetary wheel of the second stage, and the inner gear ring (i = 1, 2, N), respectively; *R*_*bs*_, *R*_*bp*1*i*_, *R*_*bp*2*i*_, and *R*_*br*_ are the radii of the base circle of the sun wheel, the ith planet wheel of the first stage, the ith planet wheel of the second stage, and the inner gear ring (i = 1, 2, N), respectively; $\overset{\cdot }{\left(\right)}$ is the derivative with respect to time; the input torque *T*_*D*_(*t*) is the fluctuation value that can be expressed as *T*_*D*_(*t*) = *T*_*D*m_ + *T*_*D*aT_(*t*), where *T*_*D*m_ is the average torque value, and *T*_*D*aT_(*t*) is the instantaneous fluctuation value that can be expressed as *T*_*D*aT_(*t*) = *T*_*D*aT_sin(*ω*_aT_*t*+*ϕ*_aT_). Parameter *e*_*sp*1*i*_(*t*) is the static transfer error between the solar wheel and the ith planetary wheel of the first stage. Fabrication and assembly of the gear can be regarded as *e*_*sp*1*i*_(*t*) = *ê*sin(*ω*_e_*t*+*ϕ*_e_). Lastly, *T*_*L*_ is the load torque applied to the output shaft of the transmission system.

Let $x_{s}=R_{bs}\theta_{s},\;x_{p1i}=R_{bp1i}\theta_{p1i},\;x_{p2i}=R_{bp2i}\theta_{p2i},\;x_{r1}=R_{br}\theta_{r1}$, where *x*_*s*_, *x*_*p*1*i*_, *x*_*p*2*i*_ and *x*_*r*1_ are the equivalent line displacements of the sun wheel, the first-stage planetary gear, the second-stage planetary gear, and the inner gear ring, respectively. The dimensional motion equation of the system can be obtained via the linear variation of Eq (5):

$$\left\{ \begin{array}{l} \sum_{i=1}^{N}(C_{sp1i}({\dot{x}}_{s}-{\dot{x}}_{p1i}-{\dot{e}}_{sp1i}(t))+K_{sp1i}(x_{s}-x_{p1i}-e_{sp1i}(t)))\\ +m_{s}{\ddot{x}}_{s}=F_{D}\\ m_{p1i}{\ddot{x}}_{p1i}+C_{sp1i}({\dot{x}}_{s}-{\dot{x}}_{p1i}-{\dot{e}}_{sp1i}(t))+K_{sp1i}(x_{s}-x_{p1i}-e_{sp1i}(t))+\\ K_{p1ip2i}(x_{p1i}+x_{p2i}-e_{p1ip2i}(t))+C_{p1ip2i}({\dot{x}}_{p1i}+{\dot{x}}_{p2i}-{\dot{e}}_{p1ip2i}(t))=0\\ m_{p2i}{\ddot{x}}_{p2i}+C_{p1ip2i}({\dot{x}}_{p1i}-{\dot{x}}_{p2i}-{\dot{e}}_{p1ip2i}(t))+K_{p1ip2i}(x_{p1i}+x_{p2i}\\ -e_{p1ip2i}(t))-K_{p2ir}(x_{r1}-x_{p2i}-e_{r1p2i}(t))-C_{p2ir}({\dot{x}}_{r1}-{\dot{x}}_{p2i}\\ -{\dot{e}}_{rp2i}(t))=0 \end{array}\right.$$ (6)

*m*_*s*_, *m*_*r*_, *m*_*p*1*i*_, *m*_*p*2*i*_ are the equivalent masses of the solar wheel, the inner ring gear, the first stage planetary wheel, and the second stage planetary wheel on the meshing line *m* = *J*/*R*^2^.

The transmission error of the gear pair X is introduced via Eq (7), where *X*_*sp*1*i*_, *X*_*rp*2*i*_, and *X*_*p*1*ip*2*i*_, represent relative displacements between the solar wheel and the first planetary wheel, between the inner gear ring and the second planetary wheel, and between the first planetary wheel and the second planetary wheel, respectively.


$$\{\begin{array}{l} X_{sp1i}=x_{s}-x_{p1i}-e_{sp1i}(t)\\ X_{rp2i}=x_{r}-x_{p2i}-e_{r1p2i}(t)\\ X_{p1ip2i}=x_{p1i}+x_{p2i}-e_{p1ip2i}(t) \end{array}$$ (7)

A dimensionless time scale *τ* = *tω*_*n*_ and displacement scale *b*_*c*_ are defined as follows: $\omega_{n}=\sqrt{K_{sp1i}(r_{bs}^{2}/J_{s}+r_{bp1i}^{2}/J_{p1i})}{,\;X}_{sp1i}=b_{c}{\bar{q}}_{1}$, $X_{rp2i}=b_{c}{\bar{q}}_{2}$, $X_{p1ip2i}=b_{c}{\bar{q}}_{3}$; $\omega_{e}={2\pi f}_{m},\omega_{h}=\omega_{e}/\omega_{n}$, *ω*_*T*_ = *ω*_*aT*_/*ω*_*n*_. Eq (6) can lead to a dimensionless equation, as shown in Eq (8):

$$\{\begin{array}{l} {\ddot{q}}_{1}-P_{s}+\sum_{i=1}^{N}\delta_{11i}{\dot{q}}_{1}+\sum_{i=1}^{N}K_{11i}f(q_{1})+\delta_{13i}{\dot{q}}_{1}+\delta_{14i}{\dot{q}}_{3}+K_{12i}f(q_{1})\\ +K_{13i}f(q_{3})=-{\ddot{e}}_{sp1i}(t)\\ {\ddot{q}}_{2}-P_{r1}+\sum_{i=1}^{N}\delta_{15i}{\dot{q}}_{2}+\sum_{i=1}^{N}K_{14i}f(q_{2})-\delta_{16i}{\dot{q}}_{3}+\delta_{17i}{\dot{q}}_{2}-K_{15i}f(q_{3})\\ +K_{16i}f(q_{2})=-{\ddot{e}}_{r1p1i}(t)\\ {\ddot{q}}_{3}+\delta_{13i}{\dot{q}}_{1}+\delta_{14i}{\dot{q}}_{3}+K_{12i}f(q_{1})+K_{13i}f(q_{3})+\delta_{16i}{\dot{q}}_{3}-\delta_{17i}{\dot{q}}_{2}\\ +K_{15i}f(q_{3})-K_{16i}f(q_{2})=-{\ddot{e}}_{p1ip2i}(t) \end{array}$$ (8)

Where

$P_{s}=\frac{F_{S}}{m_{s}b_{c}\omega_{n}^{2}}$; $\delta_{11i}=\frac{C_{sp1i}}{m_{s}\omega_{n}}$; $K_{11i}=\frac{K_{sp1i}}{m_{s}{\omega_{n}}^{2}}$; $\delta_{13i}=\frac{C_{sp1i}}{m_{p1i}\omega_{n}}$; $\delta_{14i}=\frac{C_{p1ip2i}}{m_{p1i}\omega_{n}}$; $K_{12i}=\frac{K_{sp1i}}{m_{p1i}{\omega_{n}}^{2}}$; $K_{13i}=\frac{K_{p1ip2i}}{m_{p1i}{\omega_{n}}^{2}}$; $P_{r1}=\frac{F_{L}}{m_{r1}b_{c}\omega_{n}^{2}}$; $\delta_{15i}=\frac{C_{p2ir1}}{m_{r1}\omega_{n}}$; $K_{14i}=\frac{K_{p2ir1}}{m_{r1}{\omega_{n}}^{2}}$; $\delta_{16i}=\frac{C_{p1ip2i}}{m_{p2i}\omega_{n}}$; $\delta_{17i}=\frac{C_{p2ir1}}{m_{p2i}\omega_{n}}$; $K_{15i}=\frac{K_{p1ip2i}}{m_{p2i}{\omega_{n}}^{2}}$; $K_{16i}=\frac{K_{p2ir1}}{m_{p2i}{\omega_{n}}^{2}}$.

Where the dimensionless integrated meshing error can be expressed as:

$$\left\{ \begin{array}{l} {\bar{e}}_{sp1i}(\tau )=\frac{A_{sp1i}}{b_{c}}\sin(\Omega \tau +\beta_{sp1i})\\ {\bar{e}}_{r1p1i}(\tau )=\frac{A_{r1p1i}}{b_{c}}\sin(\Omega \tau +\beta_{r1p1i})\\ {\bar{e}}_{r2p2i}(\tau )=\frac{A_{r2p2i}}{b_{c}}\sin(\Omega \tau +\beta_{r2p2i}) \end{array}\right.$$ (9)

The non-dimensional gap nonlinear function can be expressed as:

$$f(\bar{X},\bar{b})=\{\begin{array}{c} \bar{X}-\bar{b},\\ 0\\ \bar{X}+\bar{b}, \end{array}\;\begin{array}{c} \bar{X}>\bar{b}\\ |\bar{X}|\leq \bar{b}\\ \bar{X}<-\bar{b} \end{array}$$ (10)

## Low gear dynamic response of a two-speed transmission system

The low-speed gear model parameters of a two-speed planetary gear drive are shown in Table 1. The fourth-order variable step size Runge-Kutta method is used to solve Eq (8), and the initial value is q1(0) = 0, q2(0) = 0, q3(0) = 0; q1’(0) = 0, q2’(0) = 0, q3’(0) = 0. The solution interval is [0, 6000]. The excitation frequency is taken as the control parameter. Then, the transfer error bifurcation characteristics between the sun wheel and the first-stage planetary wheel, the first-stage planetary wheel and the second-stage planetary wheel, and the second-stage planetary wheel and the inner gear ring are investigated. Simultaneously, the transfer error bifurcation characteristics of the solar wheel and the first planetary wheel under different gear moduli are explored.

**Table 1:** Low-speed gear model parameters of a two-speed planetary gear transmission.

The parameter name	Parameters of the code	The parameter value
Moment of inertia of the sun wheel	*J*_*s*_	0.000534 kg.m^2^
Moment of inertia of the first planetary wheel	*J*_*p*1_	0.0000235 kg.m^2^
Moment of inertia of the second planetary wheel	*J*_*p*2_	0.0000153 kg.m^2^
Moment of inertia of the inner ring	*J*_*r*_	0.003832 kg.m^2^
The meshing damping ratio of each gear pair	*δ*	0.07
Planetary rotation number	*N*	6
backlash	*b*	0.00005 m
modulus	*m*	1.75
Solar wheel mass	*m*_*s*_	0.590 kg
The mass of the first planetary wheel	*m*_*p*1*i*_	0.135 kg
The mass of the second planetary wheel	*m*_*p*2*i*_	0.096 kg
Inner ring quality	*m*_*r*1_	0.775 kg
Tooth number of the sun wheel	*Z*_*s*_	54
Number of teeth of the first planetary gear	*Z*_*p*1*i*_	21
Number of teeth of the second planetary gear	*Z*_*p*2*i*_	19
Number of inner ring teeth	*Z*_*r*_	108
Degree of interaction between the solar wheel and the first planetary wheel	*ε*_*sp*1*i*_	1.6684
Degree of interaction between the first and second planetary wheels	*ε*_*p*1*ip*2*i*_	1.5564
Degree of contact between the second planetary wheel and the inner ring gear	*ε*_*p*2*ir*_	1.8154
The solar wheel input force	*F*_*S*_	3.6959×10^4^N
Inner ring load	*F*_*r*_	4.0849×10^4^N

### A change in transmission error between the sun and the first planetary gear with excitation frequency ω_*h*_

**Fig 3:**
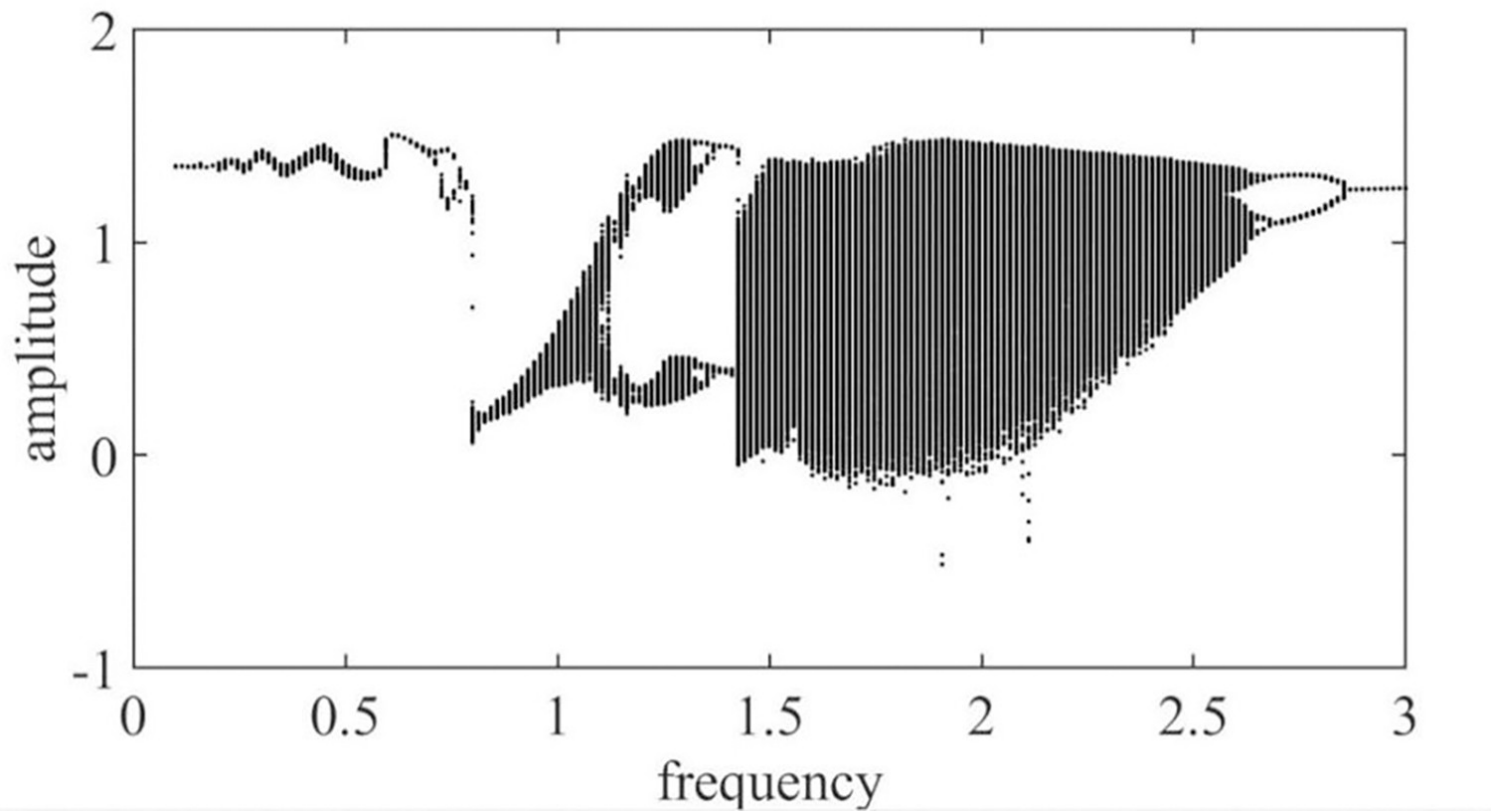
The sun and the first planetary gear with respect to the excitation frequency ω_*h*_ global bifurcation diagram.

The excitation frequency ω_*h*_ is taken as the control parameter, and the dimensionless excitation frequency is increased from 0.000 to 3.000. Then, a dimensionless dynamic transfer error bifurcation diagram of a pair of solar wheels and the first-stage planetary gear train is obtained, as shown in Fig 3.

As shown in Fig 3, the motion state of the system exhibits rich bifurcation characteristics with frequency changes: when ω_*h*_≤1.19, 2.78≤ω_*h*_≤3, the system is in a period-1 motion; when 1.19≤ω_*h*_≤1.41, 2.48≤ω_*h*_≤2.78, the system is in a multiperiodic motion; at 1.41≤ω_*h*_≤2.48, the system is in a chaotic motion.

**Fig 4:**
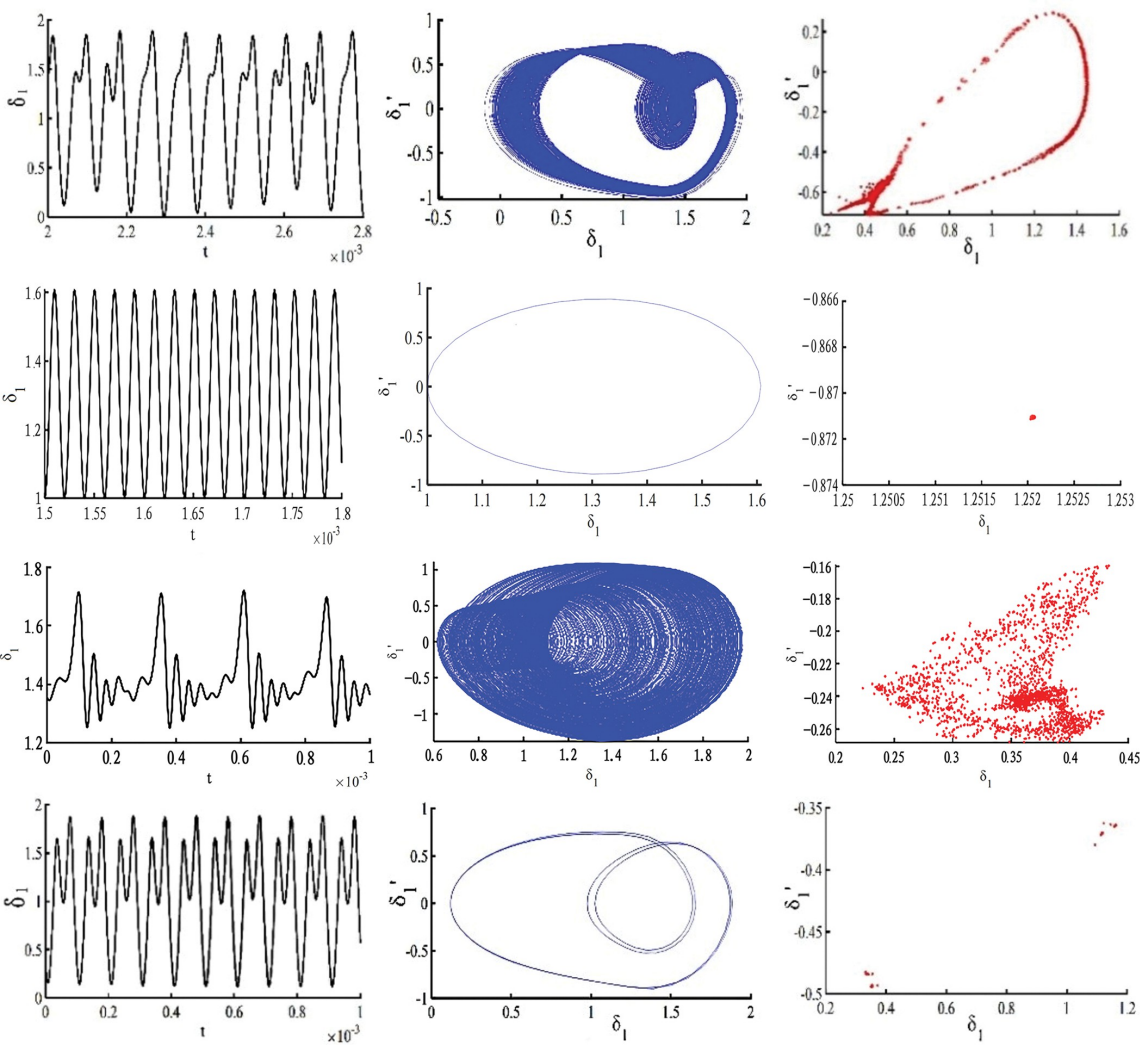
Time domain diagram, phase diagram, and Poincare cross-section diagram corresponding to different excitation frequency points of the solar wheel and the first stage planetary gears: (a) 0.63, (b) 1.03, (c) 1.92, (d) 2.71.

It can be clearly seen in spinning Figs 3 and 4 that there is a significant jump at ω_*h*_ = 0.62 and ω_*h*_ = 0.78, and at 0.62≤ω_*h*_≤ 0.78, the gear pair is in a quasi-periodic motion. Take ω_*h*_ = 0.63, the quasi-periodic motion is formed by the combination of two or more non-reducible frequencies, the phase diagram is a closed curve band with a certain width, the corresponding Poincare section is a closed curve, and its time domain diagram, phase diagram, and Poincare cross-section are shown in Fig 4(A); When the ω_*h*_≤ 0.62, the gear pair will increase slightly with the increase of the excitation frequency ω_*h*_; when 0.78≤ω_*h*_≤1.19, the gear pair increases significantly with the increase of the excitation frequency ω_*h*_, and there is a tendency to enter chaotic motion, but it is still in the period-1 motion. Take ω_*h*_ = 1.03, the phase plane diagram of the period-1 motion is a closed circle, the Poincare section is a single point, and its time domain diagram, phase diagram, and Poincare section are shown in Fig 4(B); When 1.19≤ω_*h*_≤1.41, the motion of the gear pair is period-2 motion; when the excitation frequency is located at 1.41≤ω_*h*_≤2.48, the gear pair enters chaotic motion; at this time, take ω_*h*_ = 1.92, as shown in Fig 4(C), the time domain response diagram shows aperiodic motion, the phase plane diagram is disordered, and the Poincare diagram shows many discrete points; When the excitation frequency is located at 2.48≤ω_*h*_≤2.78, the approximate motion of the system changes from period-2 motion to period-1 motion; at this time, ω_*h*_ = 2.71, as shown in Fig 4(D), the phase plane is two closed circles, and the Poincare section is approximately two single points. Finally, when the excitation frequency is 2.78≤ω_*h*_, the gear pair moves in a period-1 motion.

### The first and second planetary gears’ transmission error with respect to the excitation frequency ω_*h*_

**Fig 5:**
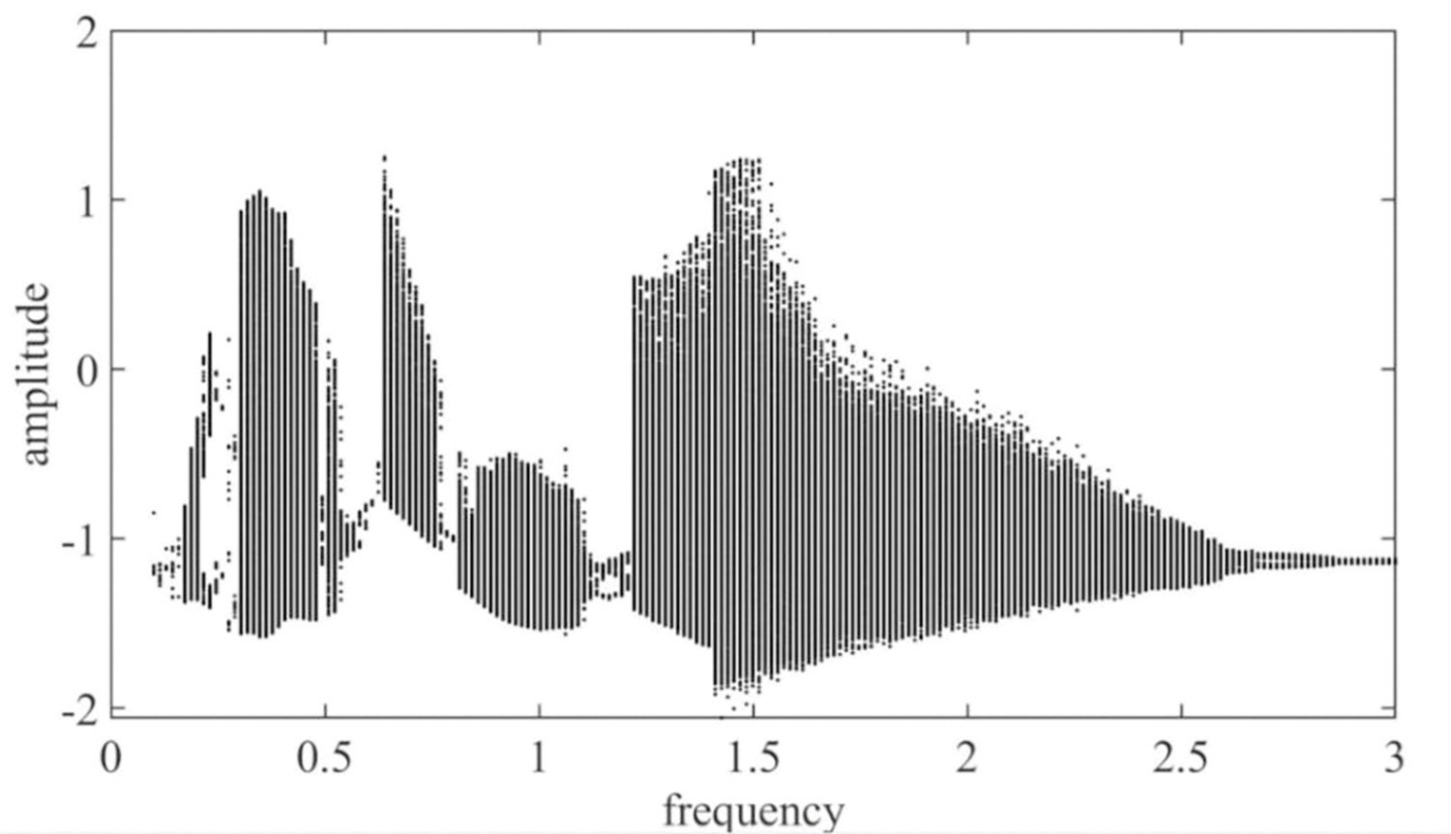
A change in the amplitude of first and second planetary gears with respect to the excitation frequency ω_*h*_.

Fig 5 shows the dimensionless transmission error bifurcation diagram of the first stage planetary gear and the second planetary gear pair drawn with the excitation frequency ω_*h*_ as the control variable in the low-speed mode of two-speed planetary gear transmission. From Fig 5, it can be seen that there are obvious jumping phenomena at ω_*h*_ = 0.28, ω_*h*_ = 0.49, ω_*h*_ = 0.61, and ω_*h*_ = 0.72, respectively. When the two-speed planetary gear transmission is located at 0.10≤ω_*h*_≤0.18, the motion does not change much; in the low frequency band 0.58≤ω_*h*_≤0.61 and 0.75≤ω_*h*_≤0.77 and high frequency band 2.88≤ω_*h*_, the system has a period-1 motion; the gear pair has a period-2 motion in the system in the low frequency band 1.18≤ω_*h*_≤1.22 and the high frequency band 2.68≤ω_*h*_≤2.79; the gear pair has a chaotic motion in the low frequency band ω_*h*_≤0.75, except for four jump points At 0.77≤ω_*h*_≤1.18 and 1.22≤ω_*h*_ in the middle and high frequency bands, ω_*h*_≤2.68 exhibit chaotic motion; at 2.79≤ω_*h*_≤3.0, the system turns to period-1 motion.

**Fig 6:**
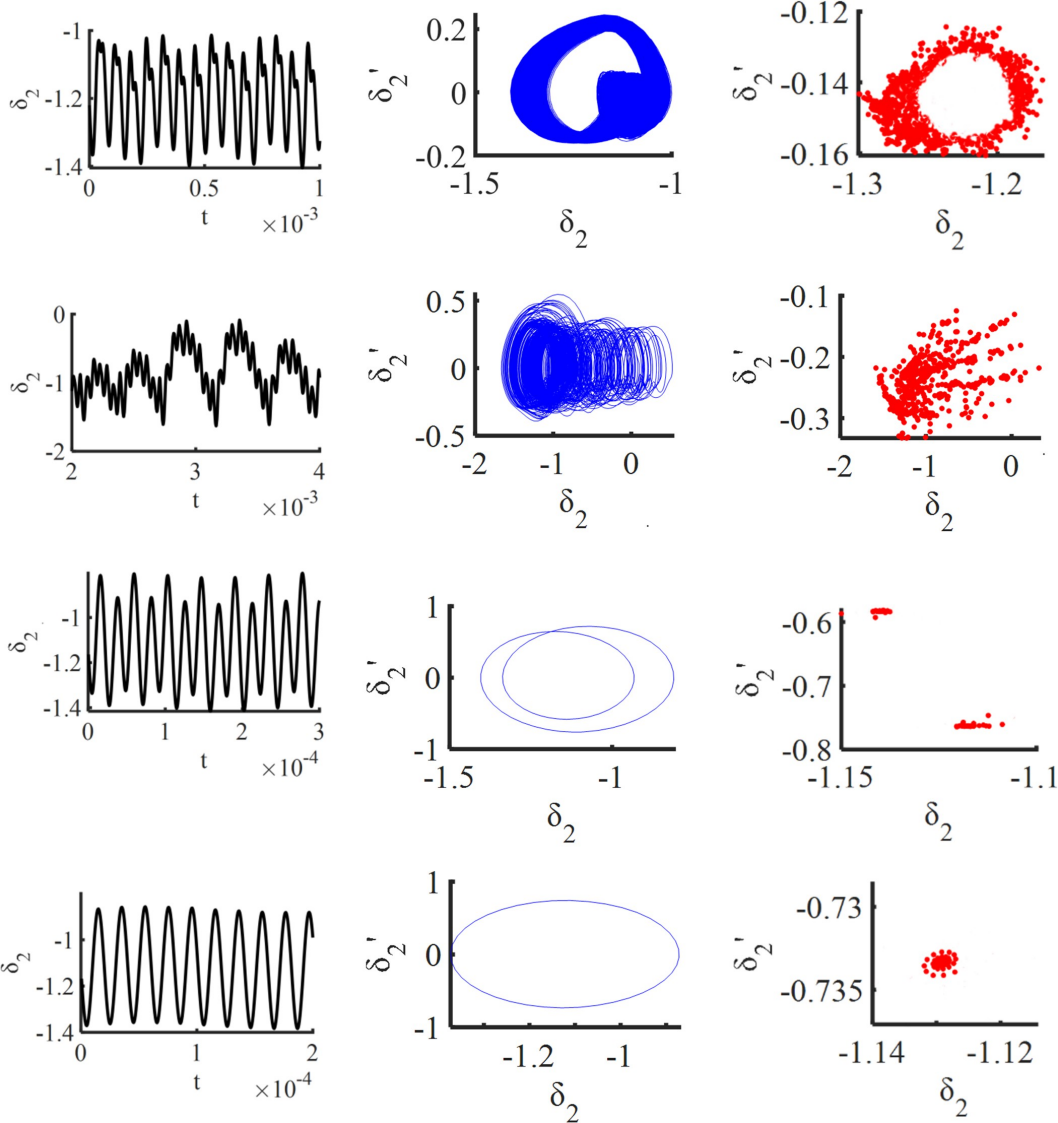
Time domain phase diagram and Poincare cross section diagram corresponding to different excitation frequency points of the first stage and second stage planetary gears: (a) 0.78, (b) 1.55, (c) 2.70, (d) 2.92.

It is clear from Figs 5 and 6 that when the frequency is small, ω_*h*_< 0.75, the system is mostly in a chaotic motion, but there are several jumps in this chaotic motion, such as at ω_*h*_ = 0.28, ω_*h*_ = 0.49, ω_*h*_ = 0.61, and ω_*h*_ = 0.72, indicating that the system vibrates during the frequency increase. With the slow increase of frequency ω_*h*_, at 0.75≤ω_*h*_≤0.77, it enters a period-1 motion; when ω_*h*_ = 0.78, the system suddenly jumps from a quasi-periodic motion state to a chaotic motion, which presents a "cataclysm" feature, indicating that the system enters the chaotic motion through the "cataclysm" pathway, as shown in Fig 5(A), because the quasi-periodic motion is formed by the combination of two or more non-reducible frequencies, and its phase diagram is a closed curve band with a certain width. The corresponding Poincare section is a closed curve. The system was in a chaotic state at 0.77≤ω_*h*_≤1.18 and 1.22≤ω_*h*_≤2.68, and ω_*h*_ = 1.55 was selected, and its time domain diagram, phase diagram, and Poincare cross-sectional diagram are shown in Fig 5(B). At 1.18≤ω_*h*_≤1.22 and 2.68≤ω_*h*_≤2.79 are in a period-2 motion, ω_*h*_ = 2.70, and the time domain diagram, phase diagram, and Poincare cross-sectional diagram are shown in Fig 5(C); the system returns to period-1 motion at 2.88≤ω_*h*_, and ω_*h*_ = 2.92 is selected, and its time domain diagram, phase diagram, and Poincare cross-sectional diagram are shown in Fig 5(D).

### A change in the transmission error for a secondary planet gear and internal gear ring with excitation frequency ω_*h*_

**Fig 7:**
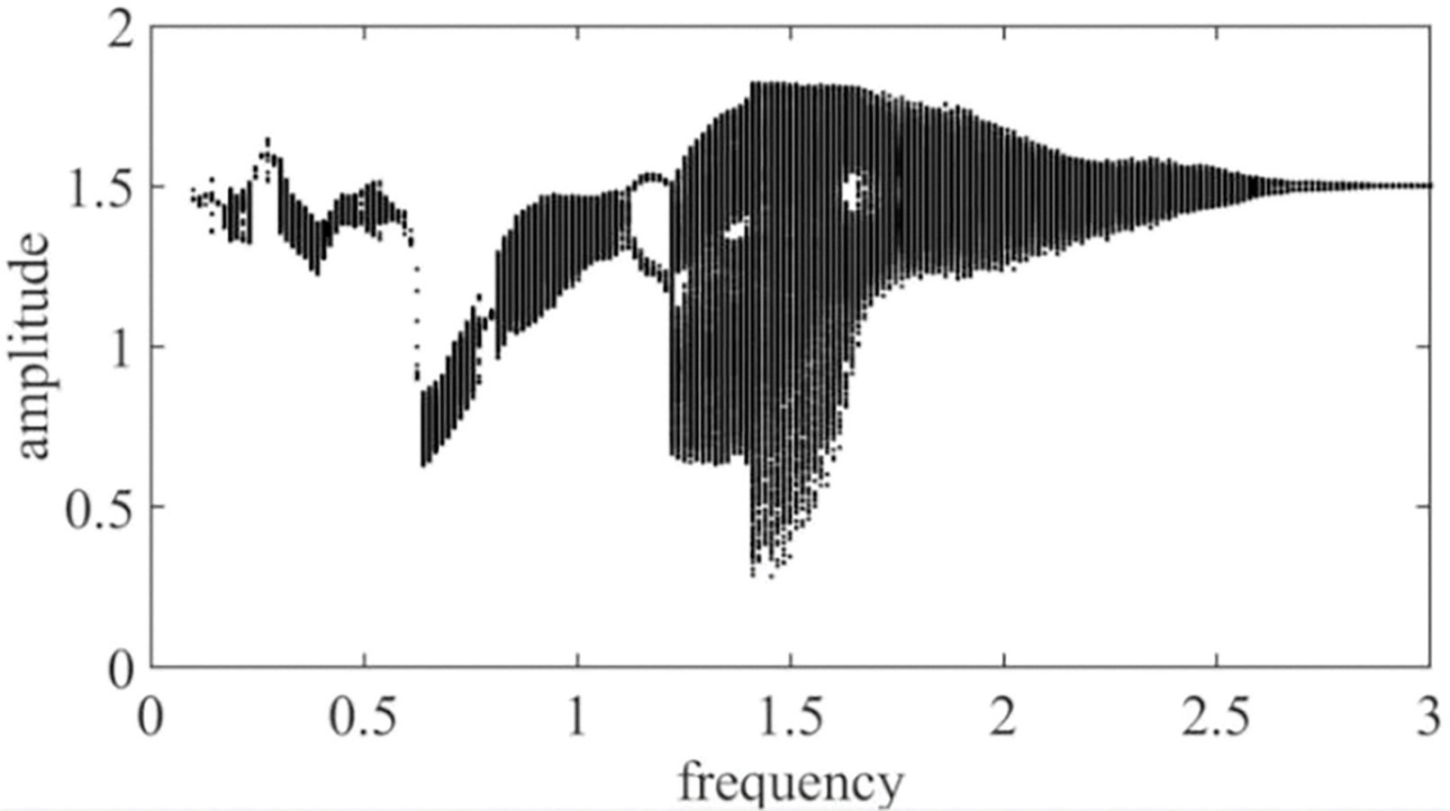
A change in the transmission error for a secondary planet gear and internal gear ring with excitation frequency ω_*h*_.

Fig 7 shows the dimensionless transmission error bifurcation diagram of the second stage planetary gear and the inner ring gear pair drawn in the low-speed gear mode of two-speed planetary gear transmission with the excitation frequency ω_*h*_ as the control variable. It can be seen that there is an obvious jump phenomenon at ω_*h*_ = 0.61 of the gear pair. When the two-speed planetary gear transmission is ω_*h*_≤0.29, the motion response of the two-speed planetary gear transmission does not change much; in the low frequency band 0.29≤ω_*h*_≤0.61, the middle and low frequency band 0.79≤ω_*h*_≤0.81 and the high frequency band 2.91≤ω_*h*_ range, the system has a period-1 motion; in the middle frequency band 1.14≤ω_*h*_≤1.21 and the high frequency band 2.71≤ω_*h*_≤2.91, there is a period-2 motion; in the 0.61≤ω_*h*_≤0.79, 0.81≤ω_*h*_≤1.14 and 1.21≤ω_*h*_≤2.71, there is chaotic motion.

**Fig 8:**
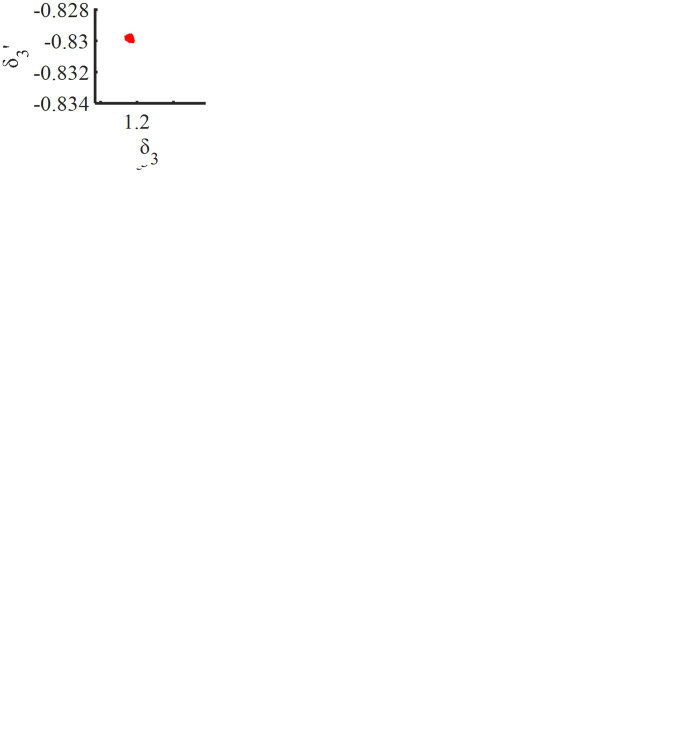
Time domain diagram, phase diagram, and Poincare cross-section diagram corresponding to different excitation frequency points of transmission errors of the second stage planetary gear and inner ring gears: (a) 0.51, (b) 1.80, (c) 2.64, (d) 2.95.

It can be clearly seen from Figs 7 and 8 that when the frequency is small, ω_*h*_≤ 0.61, the system is in an unstable period-1 motion. Take ω_*h*_ = 0.5, and its time domain diagram, phase diagram, and Poincare cross-sectional diagram are shown in Fig 8(A); With the slow increase in frequency ω_*h*_, a jump occurs at ω_*h*_ = 0.61, and the system suddenly jumps from a period-1 motion to a chaotic motion, which shows the characteristics of "cataclysm", indicating that the system enters the chaotic state through the "cataclysm" pathway. The system is in period-1 motion at 0.79≤ω_*h*_≤0.81, 0.81≤ω_*h*_≤1.14 in chaotic motion, period-2 motion at 1.14≤ω_*h*_≤1.21, and chaotic motion at 1.21≤ω_*h*_≤2.71, taking ω_*h*_ = 1.80, and its time domain diagram, phase diagram, and Poincare cross-sectional diagram are shown in Fig 8(B). At 2.71≤ω_*h*_≤2.91, the system enters a period-2 motion, taking ω_*h*_ = 2.64, and its time domain diagram, phase diagram, and Poincare cross-sectional diagram are shown in Fig 8(C); at 2.91≤ω_*h*_, it returns to a stable period-1 motion, taking ω_*h*_ = 2.95, and its time domain diagram, phase diagram, and Poincare cross-sectional diagram are shown in Fig 8(D).

### Influence of gear modulus on the transfer error bifurcation between the solar gear and the first stage planetary gear

**Fig 9:**
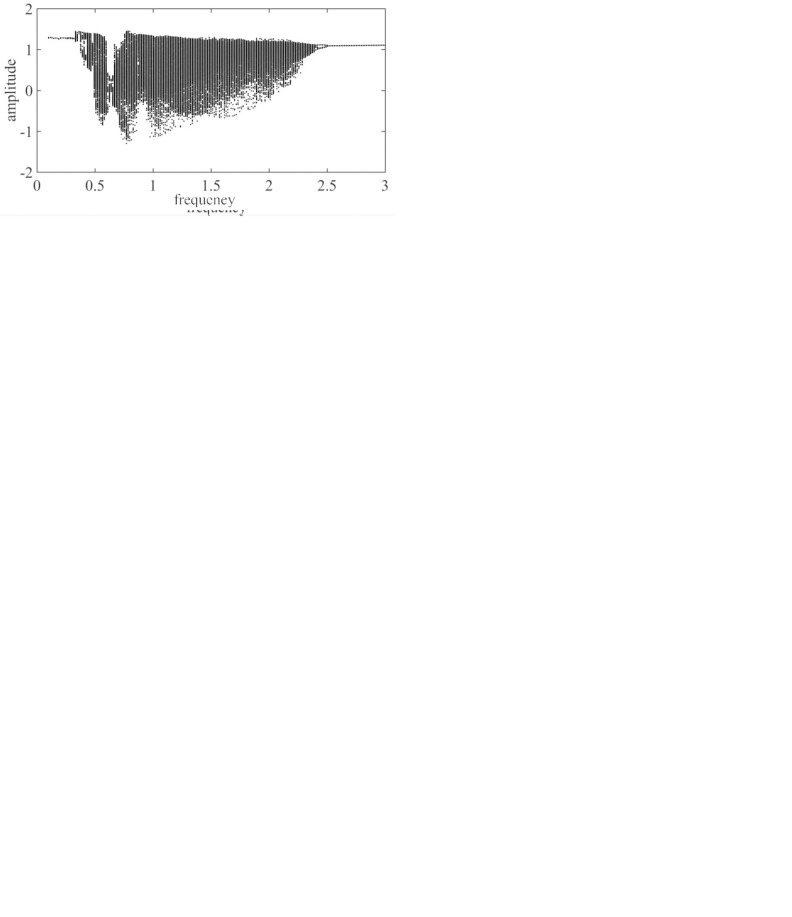
Influence of tooth modulus on transmission error bifurcation between the solar gear and the first planetary gear pair: (a) m = 1.5, (b) m = 2, (c) m = 2.5, (d) m = 3, (e) m = 4, (f) m = 5, (g) m = 6, (h) m = 8.

Fig 9 shows the bifurcation characteristics of the gear pair formed by the sun gear and the first-stage planetary gear under the condition that the gear module m is different. With the gradual increase of modulus, the system vibration amplitude difference will first briefly rise, as shown in Fig 9(A)–9(C), and the subsequent overall trend of vibration amplitude difference is gradually contracted, as shown in Fig 9(D)–9(H); From Fig 9, it can be seen that the system has experienced quasi-periodic, period-1, period-2, chaotic, and multi-periodic motions over the entire excitation frequency range.

It can be seen from Fig 9(A) that when the system is in the low excitation frequency range, the motion amplitude of the system is not large, and then there will be an obvious jumping phenomenon, gradually entering the chaotic motion; Fig 9(B)–9(H) shows that the system will gradually enter the chaotic motion prematurely with the increase of gear modulus and then end the chaotic motion prematurely. The chaotic motion is not rich enough to respond to each other and other types of motion characteristics, but with the increase in gear modulus, the fluctuation amplitude of the system motion can be suppressed to a certain extent.

Therefore, from the overall perspective of Fig 9, with the increase in modulus, the vibration amplitude difference of the system can be suppressed to a certain extent. At the same time, the excitation frequency required for the system response to enter the chaotic motion will gradually decrease with the increase in gear modulus, and the excitation frequency range span of the chaotic motion of the system will gradually decrease, and finally it will transition into a period-1 motion through a period-2 motion in the chaotic motion.

## Conclusions

Using the centralized parameter method, a six-degree-of-freedom pure writhing mechanical model of a two-speed transmission system with low-speed gear was created in this research. The model takes into account mesh stiffness, damping, tooth clearance, and overall gear error. The Runge-Kutta method was used to numerically solve the nonlinear system, with the excitation frequency serving as the control parameter. To investigate the transfer error variation between the solar wheel and the first planetary wheel, the first planetary wheel and the second planetary wheel, and the second planetary wheel and the inner ring, the global bifurcation, time domain diagram, phase diagram, and Poincare cross-section were utilized. Furthermore, the transfer error bifurcation features of the solar wheel and the first planetary wheel were thoroughly explored under different gear moduli, and the following findings were reached:

1. The two-speed transmission system has a rich cycle conversion phenomenon in the process of increasing the excitation frequency in the low-speed gear mode, which is accompanied by quasi-periodic motion, period-1 motion, period-2 motion, multi-periodic motion, and chaotic motion, but eventually converges to period-1 motion.
2. The increase in gear modulus will advance the chaotic motion, and the transformation phenomenon of motion characteristics will gradually become less obvious. In the low frequency band (ω_*h*_≤0.5), when the gear modulus is relatively small, it is generally accompanied by the jumping phenomenon, and the conversion frequency between various motion responses is higher, so it is relatively better to use high module gears in the low frequency band. When the excitation frequency is in the medium frequency band (0.5<ω_*h*_≤2), the chaotic motion of high modulus gear is too concentrated, and most of them are maintained in the high amplitude difference range, which is not conducive to the service life of the gear, and the long working time will also increase the relative error between parts, which is not conducive to the transmission of torque. There are hidden dangers to safety performance, so in the medium frequency band, it is more reasonable to use relatively low module gears when meeting the transmission torque requirements; When the excitation frequency is a high frequency band (2<ω_*h*_≤3), the amplitude difference of the three pairs of gear pairs tends to be stable, and the gear modulus has little effect on its change, so in the high frequency segment, the modulus size can be reasonably selected according to the use requirements.

## Limitations and deficiencies

1. This paper adopts the lumped parameter method in modeling and the Runge-Kutta method in solving differential equations, which is a commonly used modeling and solving method for system dynamics. However, the lumped parameter method is a simplified modeling method, which usually regards the shaft as a point mass or a rigid connection, and has certain limitations in capturing the deflection and deformation of the shaft.
2. In future studies, under the condition that the amount of calculation is appropriate, if the accuracy is required to be high, the potential energy method or Timoshenko theory can be considered for modeling, which will make the results more accurate.

## Supporting information

S1 File (DOCX)

## References

[1] Stevens MA, Lewicki DG, Handschuh RF. Concepts for multi-speed rotorcraft drive system-status of design and testing at NASA GRC. American Helicopter Society (AHS) Annual Forum. 2015.

[2] Stevens MA, Handschuh RF, Lewicki DG. Concepts for variable/multi-speed rotorcraft drive system. 64th Annual Forum and Technology Display (American Helicopter Society Forum). 2008.

[3] DeSmidt H, Wang K-W, Smith EC, Lewicki DG. Variable-Speed Simulation of a Dual-Clutch Gearbox Tiltrotor Driveline. 67th Annual Forum and Technology Display (Forum 67). 2012.

[4] Wei J, Zhang A, Qin D, Lim TC, Shu R, Lin X, et al. A coupling dynamics analysis method for a multistage planetary gear system. Mechanism and Machine Theory. 2017;110:27–49. doi: 10.1016/j.mechmachtheory.2016.12.007

[5] Qilin Huang YW, Zhipu Huo, Yudong Xie. Nonlinear Dynamic Analysis and Optimization of Closed-Form Planetary Gear System. Mathematical Problems in Engineering. 2013;1–12. doi: 10.1155/2013/149046

[6] Gui Y, Han Q, Li Z, Chu F. Detection and localization of tooth breakage fault on wind turbine planetary gear system considering gear manufacturing errors. Shock and Vibration. 2014;1–13.

[7] Lin J, Parker R. Planetary gear parametric instability caused by mesh stiffness variation. Journal of Sound vibration. 2002;249:129–145.

[8] Chaari F, Fakhfakh T, Hbaieb R, Louati J, Haddar M. Influence of manufacturing errors on the dynamic behavior of planetary gears. International Journal of Advanced Manufacturing Technology. 2006; 27:738–746.

[9] Zhang JY, Ruan ZT, Qiu DM. Dynamic Characteristics Analysis of 2K-H Planetary Gear Reducer. Journal of Xi ’an Highway Jiaotong University. 1997;(01):85–88.

[10] Sun ZM, Shen YW, Li SY. Research on Dynamic Characteristics of Closed Planetary Gear Transmission System. Chinese Journal of Mechanical Engineering. 2002;(02):44–48+52.

[11] Sun T, Shen YW, Sun ZM, Liu JY. Nonlinear Dynamic Equation Solution and Dynamic Characteristics Analysis of Planetary Gear Transmission. Journal of Mechanical Engineering. 2002;(03):11–15.

[12] Wu SJ, Liu ZH, Wang XS, Zhu EY. Nonlinear Dynamic Characteristics of Composite Planetary Gear Transmission System Based on Harmonic Balance Method. Journal of Mechanical Engineering. 2011;47(01):55–61.

[13] Xu X, Fan X, Diao P, Liu H, Technology. An investigation on the influence of modification parameters on transmission characteristics of planetary gear system. Journal of Mechanical Science Technology. 2019;33:3105–3114.

[14] Xiang L, Gao N, Hu A. Dynamic analysis of a planetary gear system with multiple nonlinear parameters. Journal of Computational Applied Mathematics. 2018; 327:325–340.

[15] Zhou D, Zhang X, Zhang Y. Dynamic reliability analysis for planetary gear system in shearer mechanisms. Mechanism and Machine Theory. 2016;105:244–259.

[16] Li M, Xie L, Ding L. Load sharing analysis and reliability prediction for planetary gear train of helicopter. Mechanism and Machine Theory. 2017;115:97–113.

[17] Xiang L, Zhang Y, Gao N, Hu A, Xing J. Nonlinear dynamics of a multistage gear transmission system with multi-clearance. International Journal of Bifurcation Chaos. 2018;28:1850034.

[18] Zhou L, Wu SJ, Li J, Wang XS, Zhu WL, Li XY. Translational Torsion Model Building and Nonlinear Dynamic Characteristics Analysis of 2K-H Planetary Gear Train. Journal of Vibration and Shock. 2016;35:(12)71-76+116.

[19] Kumar A., Vashishtha G., Gandhi C. P., Tang H., **ang J. Sparse transfer learning for identifying rotor and gear defects in the mechanical machinery. Measurement. 2021;179:109494.

[20] Vashishtha G., Chauhan S., Kumar S., Kumar R., Zimroz R., Kumar A. Intelligent fault diagnosis of worm gearbox based on adaptive CNN using amended gorilla troop optimization with quantum gate mutation strategy. Knowledge-Based Systems. 2023; 280:110984.

[21] Zhou Y., Kumar A., Gandhi C. P., Vashishtha G., Tang H., Kundu P., et al. Discrete entropy-based health indicator and LSTM for the forecasting of bearing health. Journal of the Brazilian Society of Mechanical Sciences and Engineering. 2023;45(2):120.

[22] Vashishtha G., Chauhan S., Singh M., Kumar R. Bearing defect identification by swarm decomposition considering permutation entropy measure and opposition-based slime mould algorithm. Measurement. 2021;178:109389.

[23] Vashishtha G., Chauhan S., Kumar A., Kumar R. An ameliorated African vulture optimization algorithm to diagnose the rolling bearing defects. Measurement Science and Technology. 2022;33(7):075013.

[24] Vashishtha G., Chauhan S., Yadav N., Kumar A., Kumar R. A two-level adaptive chirp mode decomposition and tangent entropy in estimation of single-valued neutrosophic cross-entropy for detecting impeller defects in centrifugal pump. Applied Acoustics. 2022;197:108905.

