# Supplementary material for: Study on nonlinear dynamic characteristics of a two-speed transmission system at low speed
Source: PLoS One. 2024 Feb 14;19(2):e0298395. doi: 10.1371/journal.pone.0298395 (PMC10866490; doi:10.1371/journal.pone.0298395)
Supplement: S1 File — (DOCX) [file pone.0298395.s001.docx]

clear;clc;close all;

%2023.09.02

bsp11=0.00005;bp21r1=0.00005;bp11p21=0.00005;wh1=1.5;

% kc=10000;omiga=1;

param1= [bsp11 bp21r1 bp11p21 wh1];

x0 = [0,0,0,0,0,0]; %给定空间，对应有i列（dyi）

T =2*pi/wh1;

tf = [0,500*T];

opt = odeset('Abstol',1e-8);

[t,x]=ode45('a313',tf,x0,opt,param1);

bsp11=0.00005;bp21r1=0.00005;bp11p21=0.00005;wh=1.5;

% kc=10000;omiga=1;

wh1 = linspace(0.1,2,100); %生成线性向量，元素为0.1-2间隔相等的200个点

for i = 1:length(wh1) %获取wh1的长度值，然后i循环从1循环到length(wh1)

T = 2*pi/wh1(i);

param1=[bsp11 bp21r1 bp11p21 wh1(i)];

t_f = 500*T;tf=0;

t01 = 0;xx=[];xend=[];xt=[];

while tf < t_f

x0 = x(end,:);

[t,x]=ode45('a313',[t01,t01+T],x0,[],param1);

xx = [xx;x(2:end,:)]; %x为第2行以后所有元素，是x的所有量，存成xx，用于瞬态分析（响应分析）

xend = [xend;x(end,:)]; %x为最后行所有元素，是每个周期的量，用于求分叉

xt = [xt;t(2:end)]; %x为第2行所有元素，时间

t01 = t(end); %相当于t01=t(lengtht)

tf = t01;

end

resl(i).xx=xx;

resl(i).xend =xend;

resl(i).xt=xt;

i;

end

save gear_clutch_system_3DOF_f(m=5 kaefa2=omiga kaefa1) resl wh1 %向前

plot(t,x(:,1))

%2023.04.10

function dy=a313(t,y,~,param1)

%----------------------------------------------------------------系统参数

bsp11=param1(1);

bp21r1=param1(2);

bp11p21=param1(3);

wh=param1(4);

zr1=108;

zp11=21;

zp21=19;

zs=54;

B1=0.025;

B2=0.0263;

B3=0.0263;

B4=0.022;

m=1.75;

ha=1.0;

% c=0.25;

a0=20*pi/180;

Ir1=0.58827;

Ip11=0.0000235;

Ip21=0.0000153;

Is=0.000534;

% 内啮合重合度

dbp21=m*zp21*cos(a0); %%%基圆半径

dbr1=m*zr1*cos(a0);

dap21=m*zp21+2*ha*m;

dar1=m*zr1-2*m*ha+(2*ha^2*m/zr1/tan(a0));%%%齿顶圆半径

ap21=acos(dbp21/dap21);

ar1=acos(dbr1/dar1);%%%齿顶圆压力角

sigamap21r1=(1/(2*pi))*(zp21*(tan(ap21)-tan(a0))-zr1*(tan(ar1)-tan(a0)));

% 外啮合重合度

dbp11=m*zp11*cos(a0); %%%基圆半径

dbs=m*zs*cos(a0);

dap11=m*zp11+2*ha*m;

das=m*zs+2*ha*m;%%%齿顶圆半径

ap11=acos(dbp11/dap11);

as=acos(dbs/das);%%%齿顶圆压力角

sigamasp11=(1/(2*pi))*(zp11*(tan(ap11)-tan(a0))+zs*(tan(as)-tan(a0)));

dbp11=m*zp11*cos(a0); %%%基圆半径

dbp21=m*zp21*cos(a0);

dap11=m*zp11+2*ha*m; %%%齿顶圆半径

dap21=m*zp21+2*ha*m;

ap11=acos(dbp11/dap11);

ap21=acos(dbp21/dap21);%%%齿顶圆压力角

sigamap11p21=(1/(2*pi))*(zp11*(tan(ap11)-tan(a0))+zp21*(tan(ap21)-tan(a0)));

mp11=(2*Ip11)/((m*zp11/2000)^2);

mp21=(2*Ip21)/((m*zp21/2000)^2);

ms=(2*Is)/((m*zs/2000)^2);

mr1=(2*Ir1)/((m*zr1/2000)^2);

% mc=0.786;

% mc1=0.275;

% mc2=0.375;

% mo=2.505;

alfa=pi/9;

rbs=(0.5*m*zs/1000)*cos(alfa);

% rbc=0.051;

rbr1=(0.5*m*zr1/1000)*cos(alfa);

rbp11=(0.5*m*zp11/1000)*cos(alfa);

rbp21=(0.5*m*zp21/1000)*cos(alfa);

% rl=54/1000;

% n=6;

Tin=1641;

% PO=1000000;

% Nin=600;

% rbo=rbr1;

% ro=0.029;

% % kds=7.149*10^5;

% % cds=318.4;

% % Tl=500;

% % Td=Tl*zs/zr1;

% n=6;

% fr1*rbr1+fcl*rbc=PO/(no*2*pi)

fs=Tin/rbs;

TL=Tin*21*108/19/54;

fr1=TL/rbr1;

% fc=fs*rbs*cos(alfa)-fr1*rbr1*cos(alfa);

% fl=TL/rl;

% fc=5936;%%假设

% fl=54881;%%假设

% fs=Tin/rbs;

% fr1=fs/2;

% TL=Tin*21*108/19/54;

% fr1=TL/rbr1;

% fc=5936;%%假设

% fl=54881;%%假设

p=pi*m;

E=2.06*10^11;

G=7.94*10^10;

rh1=0.016;%轮毂

rh2=0.011;

ha=m;%齿顶高

hf=1.25*m;%齿根高

kt1=E*p^3*B2/(32*cos(alfa)*(ha+hf)^3);

kb1=4*G*pi*rh1^2*B1/(rbs^2-rh1^2);

kb2=4*G*pi*rh2^2*B2/(rbp11^2-rh2^2);

kmin1=1/(1/kb1+2/kt1+1/kb2);

kmax1=1/(1/kb1+1/kt1+1/kb2);

k01=(2-sigamasp11)*kmin1+(sigamasp11-1)*kmax1;

ks11=(kmax1-kmin1)*(1-cos(2*pi*(sigamasp11-2)))/(pi);

ks21=(kmax1-kmin1)*(1-cos(2*pi*(sigamasp11-2)*2))/(2*pi);

ks31=(kmax1-kmin1)*(1-cos(2*pi*(sigamasp11-2)*3))/(3*pi);

ks41=(kmax1-kmin1)*(1-cos(2*pi*(sigamasp11-2)*4))/(4*pi);

ks51=(kmax1-kmin1)*(1-cos(2*pi*(sigamasp11-2)*5))/(5*pi);

kc11=(kmax1-kmin1)*(sin(2*pi*(sigamasp11-2)))/(pi);

kc21=(kmax1-kmin1)*(sin(2*pi*(sigamasp11-2)*2))/(2*pi);

kc31=(kmax1-kmin1)*(sin(2*pi*(sigamasp11-2)*3))/(3*pi);

kc41=(kmax1-kmin1)*(sin(2*pi*(sigamasp11-2)*4))/(4*pi);

kc51=(kmax1-kmin1)*(sin(2*pi*(sigamasp11-2)*5))/(5*pi);

kkh1=k01+(ks11*sin(wh*t)+kc11*cos(wh*t))+(ks21*sin(2*wh*t)+kc21*cos(2*wh*t))+(ks31*sin(3*wh*t)+kc31*cos(3*wh*t))+(ks41*sin(4*wh*t)+kc41*cos(4*wh*t))+(ks51*sin(5*wh*t)+kc51*cos(5*wh*t));

% -----------------------------------------------------------------第二对齿轮时变啮合刚度

p=pi*m;

E=2.06*10^11;

G=7.94*10^10;

rh2=0.011;%轮毂

rh3=0.011;

ha=m;%齿顶高

hf=1.25*m;%齿根高

kt3=E*p^3*B3/(32*cos(alfa)*(ha+hf)^3);

kb3=4*G*pi*rh2^2*B2/(rbp11^2-rh3^2);

kb4=4*G*pi*rh3^2*B3/(rbp21^2-rh3^2);

kmin2=1/(1/kb3+2/kt3+1/kb4);

kmax2=1/(1/kb3+1/kt3+1/kb4);

% k02=omiga*k01;

k02=(2-sigamap11p21)*kmin2+(sigamap11p21-1)*kmax2;

ks12=(kmax2-kmin2)*(1-cos(2*pi*(sigamap11p21-2)))/(pi);

ks22=(kmax2-kmin2)*(1-cos(2*pi*(sigamap11p21-2)*2))/(2*pi);

ks32=(kmax2-kmin2)*(1-cos(2*pi*(sigamap11p21-2)*3))/(3*pi);

ks42=(kmax2-kmin2)*(1-cos(2*pi*(sigamap11p21-2)*4))/(4*pi);

ks52=(kmax2-kmin2)*(1-cos(2*pi*(sigamap11p21-2)*5))/(5*pi);

kc12=(kmax2-kmin2)*(sin(2*pi*(sigamap11p21-2)))/(pi);

kc22=(kmax2-kmin2)*(sin(2*pi*(sigamap11p21-2)*2))/(2*pi);

kc32=(kmax2-kmin2)*(sin(2*pi*(sigamap11p21-2)*3))/(3*pi);

kc42=(kmax2-kmin2)*(sin(2*pi*(sigamap11p21-2)*4))/(4*pi);

kc52=(kmax2-kmin2)*(sin(2*pi*(sigamap11p21-2)*5))/(5*pi);

% wcc=95/80;

kkh2=k02+(ks12*sin(wh*t)+kc12*cos(wh*t))+(ks22*sin(2*wh*t)+kc22*cos(2*wh*t))+(ks32*sin(3*wh*t)+kc32*cos(3*wh*t))+(ks42*sin(4*wh*t)+kc42*cos(4*wh*t))+(ks52*sin(5*wh*t)+kc52*cos(5*wh*t));

% -----------------------------------------------------------------第三对齿轮时变啮合刚度

p=pi*m;

E=2.06*10^11;

G=7.94*10^10;

rh3=0.011;%轮毂

rh4=0.011;

ha=m;%齿顶高

hf=1.25*m;%齿根高

kt5=E*p^3*B4/(32*cos(alfa)*(ha+hf)^3);

kb5=4*G*pi*rh3^2*B3/(rbp21^2-rh3^2);

kb6=4*G*pi*rh4^2*B4/(rbr1^2-rh4^2);

kmin3=1/(1/kb5+2/kt5+1/kb6);

kmax3=1/(1/kb5+1/kt5+1/kb6);

% k02=omiga*k01;

k03=(2-sigamap21r1)*kmin3+(sigamap21r1-1)*kmax3;

ks13=(kmax3-kmin3)*(1-cos(2*pi*(sigamap21r1-2)))/(pi);

ks23=(kmax3-kmin3)*(1-cos(2*pi*(sigamap21r1-2)*2))/(2*pi);

ks33=(kmax3-kmin3)*(1-cos(2*pi*(sigamap21r1-2)*3))/(3*pi);

ks43=(kmax3-kmin3)*(1-cos(2*pi*(sigamap21r1-2)*4))/(4*pi);

ks53=(kmax3-kmin3)*(1-cos(2*pi*(sigamap21r1-2)*5))/(5*pi);

kc13=(kmax3-kmin3)*(sin(2*pi*(sigamap21r1-2)))/(pi);

kc23=(kmax3-kmin3)*(sin(2*pi*(sigamap21r1-2)*2))/(2*pi);

kc33=(kmax3-kmin3)*(sin(2*pi*(sigamap21r1-2)*3))/(3*pi);

kc43=(kmax3-kmin3)*(sin(2*pi*(sigamap21r1-2)*4))/(4*pi);

kc53=(kmax3-kmin3)*(sin(2*pi*(sigamap21r1-2)*5))/(5*pi);

% wcc=95/80;

kkh3=k03+(ks13*sin(wh*t)+kc13*cos(wh*t))+(ks23*sin(2*wh*t)+kc23*cos(2*wh*t))+(ks33*sin(3*wh*t)+kc33*cos(3*wh*t))+(ks43*sin(4*wh*t)+kc43*cos(4*wh*t))+(ks53*sin(5*wh*t)+kc53*cos(5*wh*t));

% ksp11max=7.778*10^8;

% ksp11min=5.026*10^8;

% kp21r1max=2.846*10^8;

% kp21r1min=1.812*10^8;

%

% kp11p21max=2.641*10^8;

% kp11p21min=1.727*10^8;

%

% khsp1=(sigamasp11-1)*ksp11max+(2-sigamasp11)*ksp11min;

%

% khp1p2=(sigamap11p21-1)*kp11p21max+(2-sigamap11p21)*kp11p21min;

%

% khp2r1=(sigamap21r1-1)*kp21r1max+(2-sigamap21r1)*kp21r1min;

% ka1=1.32*10^5;%%假设

% ka2=1.32*10^5;

% ca1=0;%%假设

% ca2=0;

% k1=1.4776*10^4;%%假设

% c1=0;%%假设

% bp21r1=10*10^(-6);

% bp11p21=10*10^(-6);

% bsp11=10*10^(-6);

bc=1*10^(-5);

e=10*10^(-6);

wn=(k01*(rbs^2/Is+rbp11^2/Ip11))^0.5;

kesai1=0.04;

kesai2=0.04;

kesai3=0.04;

csp11=2*kesai1*(k01/(1/ms+1/mp11))^0.5;

cp11p21=2*kesai2*(k01/(1/mp11+1/mp21))^0.5;

cp21r1=2*kesai3*(k01/(1/mr1+1/mp21))^0.5;

% t=2;

% sigamasp11=param(1);

% sigamap11p21=param(2);

% sigamap21r1=param(3);

% wh=param;

% cc1=0;

% cc2=0;

%

% khsp1=(2-sigamasp11)*ksp11min+(sigamasp11-1)*ksp11max;

% khps1=(khsp1/sigamasp11)*(2-2*cos(2*pi*(sigamasp11-1)))^0.5/pi;

% faips1=atan((1-cos(2*pi*(sigamasp11-1)))/(sin(2*pi*(sigamasp11-1))));

% khps2=(khsp1/sigamasp11)*(2-2*cos(4*pi*(sigamasp11-1)))^0.5/(2*pi);

% faips2=atan((1-cos(4*pi*(sigamasp11-1)))/(sin(4*pi*(sigamasp11-1))));

% khps3=(khsp1/sigamasp11)*(2-2*cos(6*pi*(sigamasp11-1)))^0.5/(3*pi);

% faips3=atan((1-cos(6*pi*(sigamasp11-1)))/(sin(6*pi*(sigamasp11-1))));

% khps4=(khsp1/sigamasp11)*(2-2*cos(8*pi*(sigamasp11-1)))^0.5/(4*pi);

% faips4=atan((1-cos(8*pi*(sigamasp11-1)))/(sin(8*pi*(sigamasp11-1))));

% khps5=(khsp1/sigamasp11)*(2-2*cos(10*pi*(sigamasp11-1)))^0.5/(5*pi);

% faips5=atan((1-cos(10*pi*(sigamasp11-1)))/(sin(10*pi*(sigamasp11-1))));

%

% khp2r1=(2-sigamap21r1)*kp21r1min+(sigamap21r1-1)*kp21r1max;

% khpr1=(khp2r1/sigamap21r1)*(2-2*cos(2*pi*(sigamap21r1-1)))^0.5/pi;

% faipr1=atan((1-cos(2*pi*(sigamap21r1-1)))/(sin(2*pi*(sigamap21r1-1))));

% khpr2=(khp2r1/sigamap21r1)*(2-2*cos(4*pi*(sigamap21r1-1)))^0.5/(2*pi);

% faipr2=atan((1-cos(4*pi*(sigamap21r1-1)))/(sin(4*pi*(sigamap21r1-1))));

% khpr3=(khp2r1/sigamap21r1)*(2-2*cos(6*pi*(sigamap21r1-1)))^0.5/(3*pi);

% faipr3=atan((1-cos(6*pi*(sigamap21r1-1)))/(sin(6*pi*(sigamap21r1-1))));

% khpr4=(khp2r1/sigamap21r1)*(2-2*cos(8*pi*(sigamap21r1-1)))^0.5/(4*pi);

% faipr4=atan((1-cos(8*pi*(sigamap21r1-1)))/(sin(8*pi*(sigamap21r1-1))));

% khpr5=(khp2r1/sigamap21r1)*(2-2*cos(10*pi*(sigamap21r1-1)))^0.5/(5*pi);

% faipr5=atan((1-cos(10*pi*(sigamap21r1-1)))/(sin(10*pi*(sigamap21r1-1))));

%

% khp1p2=(2-sigamap11p21)*kp11p21min+(sigamap11p21-1)*kp11p21max;

% khp11p21=(khp1p2/sigamap11p21)*(2-2*cos(2*pi*(sigamap11p21-1)))^0.5/pi;

% faip1p21=atan((1-cos(2*pi*(sigamap11p21-1)))/(sin(2*pi*(sigamap11p21-1))));

% khp11p22=(khp1p2/sigamap11p21)*(2-2*cos(4*pi*(sigamap11p21-1)))^0.5/(2*pi);

% faip1p22=atan((1-cos(4*pi*(sigamap11p21-1)))/(sin(4*pi*(sigamap11p21-1))));

% khp11p23=(khp1p2/sigamap11p21)*(2-2*cos(6*pi*(sigamap11p21-1)))^0.5/(3*pi);

% faip1p23=atan((1-cos(6*pi*(sigamap11p21-1)))/(sin(6*pi*(sigamap11p21-1))));

% khp11p24=(khp1p2/sigamap11p21)*(2-2*cos(8*pi*(sigamap11p21-1)))^0.5/(4*pi);

% faip1p24=atan((1-cos(8*pi*(sigamap11p21-1)))/(sin(8*pi*(sigamap11p21-1))));

% khp11p25=(khp1p2/sigamap11p21)*(2-2*cos(10*pi*(sigamap11p21-1)))^0.5/(5*pi);

% faip1p25=atan((1-cos(10*pi*(sigamap11p21-1)))/(sin(10*pi*(sigamap11p21-1))));

%

% ksp11=(khps1+khps1*cos(wh*t-faips1)+khps2*cos(2*wh*t-faips2)+khps3*cos(3*wh*t-faips3)+khps4*cos(4*wh*t-faips4)+khps5*cos(5*wh*t-faips5));

% kp21r1=(khp2r1+khpr1*cos(wh*t-faipr1)+khpr2*cos(2*wh*t-faipr2)+khpr3*cos(3*wh*t-faipr3)+khpr4*cos(4*wh*t-faipr4)+khpr5*cos(5*wh*t-faipr5));

% kp11p21=(khp1p2+khp11p21*cos(wh*t-faip1p21)+khp11p22*cos(2*wh*t-faip1p22)+khp11p23*cos(3*wh*t-faip1p23)+khp11p24*cos(4*wh*t-faip1p24)+khp11p25*cos(5*wh*t-faip1p25));

kesi111=csp11/(ms*wn);

% kesi112=kesi111;

% kesi113=kesi112;

% kesi114=kesi113;

% kesi115=kesi114;

% kesi116=kesi115;

kesi121=csp11/(mp11*wn);

% kesi132=kesi131;

% kesi133=kesi132;

% kesi134=kesi133;

% kesi135=kesi134;

% kesi136=kesi135;

kesi131=cp11p21/(bc*mp11*wn);

% kesi142=kesi141;

% kesi143=kesi142;

% kesi144=kesi143;

% kesi145=kesi144;

% kesi146=kesi145;

kesi141=cp11p21/(mp11*wn);

% kesi152=kesi151;

% kesi153=kesi152;

% kesi154=kesi153;

% kesi155=kesi154;

% kesi156=kesi155;

kesi151=cp21r1/(mp11*wn);

% kesi162=kesi161;

% kesi163=kesi162;

% kesi164=kesi163;

% kesi165=kesi164;

% kesi166=kesi165;

kesi161=cp11p21/(mp21*wn);

% kesi172=kesi171;

% kesi173=kesi172;

% kesi174=kesi173;

% kesi175=kesi174;

% kesi176=kesi175;

kesi171=cp21r1/(mp21*wn);

% kesi20=csp11/(mc1*wn);

% kesi21=cp11p21/(mc1*wn);

% kesi22=cp21r1/(mc1*wn);

% kesi23=ca2/(mc2*wn);

% kesi24=(c1/(mr1*wn))+(c1/(mo*wn));

kapa111=kkh1/(bc*ms*wn^2);

% kapa112=kapa111;

% kapa113=kapa112;

% kapa114=kapa113;

% kapa115=kapa114;

% kapa116=kapa115;

kapa121=kkh1/(bc*mp11*wn^2);

% kapa122=kapa121;

% kapa123=kapa122;

% kapa124=kapa123;

% kapa125=kapa124;

% kapa126=kapa125;

kapa131=kkh2/(bc*mp11*wn^2);

% kapa132=kapa131;

% kapa133=kapa132;

% kapa134=kapa133;

% kapa135=kapa134;

% kapa136=kapa135;

kapa141=kkh3/(bc*mp11*wn^2);

% kapa142=kapa141;

% kapa143=kapa142;

% kapa144=kapa143;

% kapa145=kapa144;

% kapa146=kapa145;

kapa151=kkh1/(bc*mp11*wn^2);

% kapa152=kapa151;

% kapa153=kapa152;

% kapa154=kapa153;

% kapa155=kapa154;

% kapa156=kapa155;

kapa161=kkh2/(bc*mp21*wn^2);

% kapa162=kapa161;

% kapa163=kapa162;

% kapa164=kapa163;

% kapa165=kapa164;

% kapa166=kapa165;

kapa171=kkh3/(bc*mp21*wn^2);

% kapa20=ksp11/(mc1*wn^2);

% kapa21=kp11p21/(mc1*wn^2);

% kapa22=kp21r1/(mc1*wn^2);

% kapa23 =ka2/(mc2*wn^2);

% kapa24=(k1/(mr1*wn^2))+(k1/(mo*wn^2));

ps=fs/(ms*bc*wn^2);

pr1=fr1/(mp21*bc*wn^2);

% pc1=fc/(mc1*bc*wn^2);

% pr2=fc/(mc2*bc*wn^2);

% po=fl/(mo*bc*wn^2);

% f36=0;f1=0;f3=0;f5=0;f7=0;f9=0;f11=0;f13=0;f15=0;f17=0;f19=0;f21=0;f23=0;f25=0;f27=0;f29=0;f31=0;f33=0;f35=0;%ksp11=0;ksp12=0;ksp13=0;ksp14=0;ksp15=0;ksp16=0;kp21r1=0;kp22r1=0;kp23r1=0;kp24r1=0;kp25r1=0;kp26r1=0;kp11p21=0;kp12p22=0;kp13p23=0;kp14p24=0;kp15p25=0;kp16p26=0;

if y(1)>bsp11/bc

f1=y(1)-bsp11/bc;

elseif y(1)<-bsp11/bc

f1=y(1)+bsp11/bc;

else

f1=0;

end

if y(3)>bp21r1/bc

f2=y(3)-bp21r1/bc;

elseif y(3)<-bp21r1/bc

f2=y(3)+bp21r1/bc;

else

f2=0;

end

if y(5)>bp11p21/bc

f3=y(5)-bp11p21/bc;

elseif y(5)<-bp11p21/bc

f3=y(5)+bp11p21/bc;

else

f3=0;

end

% if y(7)>bsp14/bc

% f7=y(7)-bsp14/bc;

% else if y(7)<-bsp14/bc

% f7=y(7)+bsp14/bc;

% else

% f7=0;

% end

% end

%

% if y(9)>bsp15/bc

% f9=y(9)-bsp15/bc;

% else if y(9)<-bsp15/bc

% f9=y(9)+bsp15/bc;

% else

% f9=0;

% end

% end

%

% if y(11)>bsp16/bc

% f11=y(11)-bsp16/bc;

% else if y(11)<-bsp16/bc

% f11=y(11)+bsp16/bc;

% else

% f11=0;

% end

%

% if y(13)>bp21r1/bc

% f13=y(13)-bp21r1/bc;

% else if y(13)<-bp21r1/bc

% f13=y(13)+bp21r1/bc;

% else

% f13=0;

% end

% end

%

% if y(15)>bp22r1/bc

% f15=y(15)-bp22r1/bc;

% else if y(15)<-bp22r1/bc

% f15=y(15)+bp22r1/bc;

% else

% f15=0;

% end

% end

%

% if y(17)>bp23r1/bc

% f17=y(17)-bp23r1/bc;

% else if y(17)<-bp23r1/bc

% f17=y(17)+bp23r1/bc;

% else

% f17=0;

% end

% end

%

% if y(19)>bp24r1/bc

% f19=y(19)-bp24r1/bc;

% else if y(19)<-bp24r1/bc

% f19=y(19)+bp24r1/bc;

% else

% f19=0;

% end

% end

%

% if y(21)>bp25r1/bc

% f21=y(21)-bp25r1/bc;

% else if y(21)<-bp25r1/bc

% f21=y(21)+bp25r1/bc;

% else

% f21=0;

% end

% end

%

% if y(23)>bp26r1/bc

% f23=y(23)-bp26r1/bc;

% else if y(23)<-bp26r1/bc

% f23=y(23)+bp26r1/bc;

% else

% f23=0;

% end

% end

% if y(25)>bp11p21/bc

% f25=y(25)-bp11p21/bc;

% else if y(25)<-bp11p21/bc

% f25=y(25)+bp11p21/bc;

% else

% f25=0;

% end

% end

%

% if y(27)>bp12p22/bc

% f27=y(27)-bp12p22/bc;

% else if y(27)<-bp12p22/bc

% f27=y(27)+bp12p22/bc;

% else

% f27=0;

% end

% end

%

% if y(29)>bp13p23/bc

% f29=y(29)-bp13p23/bc;

% else if y(29)<-bp13p23/bc

% f29=y(29)+bp13p23/bc;

% else

% f29=0;

% end

% end

%

% if y(31)>bp14p24/bc

% f31=y(31)-bp14p24/bc;

% else if y(31)<-bp14p24/bc

% f31=y(31)+bp14p24/bc;

% else

% f31=0;

% end

% end

% if y(33)>bp15p25/bc

% f33=y(33)-bp15p25/bc;

% else if y(33)<-bp15p25/bc

% f33=y(33)+bp15p25/bc;

% else

% f33=0;

% end

% end

%

% if y(35)>bp16p26/bc

% f35=y(35)-bp16p26/bc;

% else if y(35)<-bp16p26/bc

% f35=y(35)+bp16p26/bc;

% else

% f35=0;

% end

% end

%

% if y(36)>0

% f36=y(36);

% else

% f36=0;

% end

dy=zeros(6,1);

dy(1)=y(2);

dy(2)=ps-6*kesi111*y(2)-6*kapa111*f1-kesi121*y(2)+kapa121*f1+kapa131*f3+kesi131*f2+(e/bc)*(wh^2)*sin(wh*t);

% dy(3)=y(4);

% dy(4)=ps-(kesi111+kesi112+kesi113+kesi114+kesi115+kesi116)*y(2)-(kapa111+kapa112+kapa113+kapa114+kapa115+kapa116)*y(1)-kesi132*y(2)-kesi142*y(26)-kapa122*y(1)-kapa132*y(25)+(e/bc)*(wh^2)*sin(wh*t);

% dy(5)=y(6);

% dy(6)=ps-(kesi111+kesi112+kesi113+kesi114+kesi115+kesi116)*y(2)-(kapa111+kapa112+kapa113+kapa114+kapa115+kapa116)*y(1)-kesi133*y(2)-kesi143*y(26)-kapa123*y(1)-kapa133*y(25)+(e/bc)*(wh^2)*sin(wh*t);

% dy(7)=y(8);

% dy(8)=ps-(kesi111+kesi112+kesi113+kesi114+kesi115+kesi116)*y(2)-(kapa111+kapa112+kapa113+kapa114+kapa115+kapa116)*y(1)-kesi134*y(2)-kesi144*y(26)-kapa124*y(1)-kapa134*y(25)+(e/bc)*(wh^2)*sin(wh*t);

% dy(9)=y(10);

% dy(10)=ps-(kesi111+kesi112+kesi113+kesi114+kesi115+kesi116)*y(2)-(kapa111+kapa112+kapa113+kapa114+kapa115+kapa116)*y(1)-kesi135*y(2)-kesi145*y(26)-kapa125*y(1)-kapa135*y(25)+(e/bc)*(wh^2)*sin(wh*t);

% dy(11)=y(12);

% dy(12)=ps-(kesi111+kesi112+kesi113+kesi114+kesi115+kesi116)*y(2)-(kapa111+kapa112+kapa113+kapa114+kapa115+kapa116)*y(1)-kesi136*y(2)-kesi146*y(26)-kapa126*y(1)-kapa136*y(25)+(e/bc)*(wh^2)*sin(wh*t);

dy(3)=y(4);

dy(4)=ps-pr1-6*kesi111*y(2)-6*kapa111*f1-kesi141*y(6)+kapa131*f3+kapa141*f2+kesi151*y(4)+(e/bc)*(wh^2)*sin(wh*t);

% dy(15)=y(16);

% dy(16)=pr1-(kesi151+kesi152+kesi153+kesi154+kesi155+kesi156)*y(14)-(kapa141+kapa142+kapa143+kapa144+kapa145+kapa146)*y(13)-kesi162*y(26)-kesi172*y(14)-kapa152*y(25)-kapa162*y(13)+(e/bc)*(wh^2)*sin(wh*t);

% dy(17)=y(18);

% dy(18)=pr1-(kesi151+kesi152+kesi153+kesi154+kesi155+kesi156)*y(14)-(kapa141+kapa142+kapa143+kapa144+kapa145+kapa146)*y(13)-kesi163*y(26)-kesi173*y(14)-kapa153*y(25)-kapa163*y(13)+(e/bc)*(wh^2)*sin(wh*t);

% dy(19)=y(20);

% dy(20)=pr1-(kesi151+kesi152+kesi153+kesi154+kesi155+kesi156)*y(14)-(kapa141+kapa142+kapa143+kapa144+kapa145+kapa146)*y(13)-kesi164*y(26)-kesi174*y(14)-kapa154*y(25)-kapa164*y(13)+(e/bc)*(wh^2)*sin(wh*t);

% dy(21)=y(22);

% dy(22)=pr1-(kesi151+kesi152+kesi153+kesi154+kesi155+kesi156)*y(14)-(kapa141+kapa142+kapa143+kapa144+kapa145+kapa146)*y(13)-kesi165*y(26)-kesi175*y(14)-kapa155*y(25)-kapa165*y(13)+(e/bc)*(wh^2)*sin(wh*t);

% dy(23)=y(24);

% dy(24)=pr1-(kesi151+kesi152+kesi153+kesi154+kesi155+kesi156)*y(14)-(kapa141+kapa142+kapa143+kapa144+kapa145+kapa146)*y(13)-kesi166*y(26)-kesi176*y(14)-kapa156*y(25)-kapa166*y(13)+(e/bc)*(wh^2)*sin(wh*t);

dy(5)=y(6);

dy(6)=-kesi121*y(2)+kapa151*f1-kapa131*f3-kesi141*y(6)-kesi161*y(6)+kapa161*f3+kapa171*f2+kesi171*y(4)+(e/bc)*(wh^2)*sin(wh*t);

% dy(27)=y(28);

% dy(28)=-kesi19*y(38)-kapa18*y(37)-kesi132*y(2)-kesi142*y(26)-kapa122*y(1)-kapa132*y(25)-kesi162*y(26)-kesi172*y(14)-kapa152*y(25)-kapa162*y(13)+(e/bc)*(wh^2)*sin(wh*t);

% dy(29)=y(30);

% dy(30)=-kesi19*y(38)-kapa18*y(37)-kesi133*y(2)-kesi143*y(26)-kapa123*y(1)-kapa133*y(25)-kesi163*y(26)-kesi173*y(14)-kapa153*y(25)-kapa163*y(13)+(e/bc)*(wh^2)*sin(wh*t);

% dy(31)=y(32);

% dy(32)=-kesi19*y(38)-kapa18*y(37)-kesi134*y(2)-kesi144*y(26)-kapa124*y(1)-kapa134*y(25)-kesi164*y(26)-kesi174*y(14)-kapa154*y(25)-kapa164*y(13)+(e/bc)*(wh^2)*sin(wh*t);

% dy(33)=y(34);

% dy(34)=-kesi19*y(38)-kapa18*y(37)-kesi135*y(2)-kesi145*y(26)-kapa125*y(1)-kapa135*y(25)-kesi165*y(26)-kesi175*y(14)-kapa155*y(25)-kapa165*y(13)+(e/bc)*(wh^2)*sin(wh*t);

% dy(35)=y(36);

% dy(36)=-kesi19*y(38)-kapa18*y(37)-kesi136*y(2)-kesi146*y(26)-kapa126*y(1)-kapa136*y(25)-kesi166*y(26)-kesi176*y(14)-kapa156*y(25)-kapa166*y(13)+(e/bc)*(wh^2)*sin(wh*t);

end
